# Parallel evolution of genome structure and transcriptional landscape in the Epsilonproteobacteria

**DOI:** 10.1186/1471-2164-14-616

**Published:** 2013-09-12

**Authors:** Ida Porcelli, Mark Reuter, Bruce M Pearson, Thomas Wilhelm, Arnoud HM van Vliet

**Affiliations:** 1 Gut Health and Food Safety Programme, Institute of Food Research, Colney Lane, Norwich, NR4 7UA, UK

## Abstract

**Background:**

Gene reshuffling, point mutations and horizontal gene transfer contribute to bacterial genome variation, but require the genome to rewire its transcriptional circuitry to ensure that inserted, mutated or reshuffled genes are transcribed at appropriate levels. The genomes of Epsilonproteobacteria display very low synteny, due to high levels of reshuffling and reorganisation of gene order, but still share a significant number of gene orthologs allowing comparison. Here we present the primary transcriptome of the pathogenic Epsilonproteobacterium *Campylobacter jejuni*, and have used this for comparative and predictive transcriptomics in the Epsilonproteobacteria.

**Results:**

Differential RNA-sequencing using 454 sequencing technology was used to determine the primary transcriptome of *C. jejuni* NCTC 11168, which consists of 992 transcription start sites (TSS), which included 29 putative non-coding and stable RNAs, 266 intragenic (internal) TSS, and 206 antisense TSS. Several previously unknown features were identified in the *C. jejuni* transcriptional landscape, like leaderless mRNAs and potential leader peptides upstream of amino acid biosynthesis genes. A cross-species comparison of the primary transcriptomes of *C. jejuni* and the related Epsilonproteobacterium *Helicobacter pylori* highlighted a lack of conservation of operon organisation, position of intragenic and antisense promoters or leaderless mRNAs. Predictive comparisons using 40 other Epsilonproteobacterial genomes suggests that this lack of conservation of transcriptional features is common to all Epsilonproteobacterial genomes, and is associated with the absence of genome synteny in this subdivision of the Proteobacteria.

**Conclusions:**

Both the genomes and transcriptomes of Epsilonproteobacteria are highly variable, both at the genome level by combining and division of multicistronic operons, but also on the gene level by generation or deletion of promoter sequences and 5′ untranslated regions. Regulatory features may have evolved after these species split from a common ancestor, with transcriptome rewiring compensating for changes introduced by genomic reshuffling and horizontal gene transfer.

## Background

While our appreciation of microbial diversity has been greatly increased by the exponential increase in the availability of genome sequences and by metagenomic approaches [1,2], it has also highlighted our relative lack of understanding about what drives variation, and which limitations and constraints control the process of genome variation. Diversity at the level of gene order and genome content can be introduced via the reorganisation of the genome, through combinations of gene inversion, recombination, gene duplication, deletion and horizontal gene transfer [3,4]. Such movement, deletion or introduction of genes or operons can create a problem for the cell, as the reorganisation of the genome may result in disruption of transcriptional circuitry controlling the expression levels of such genes. However, variability can also be introduced at the gene level, e.g. by generation of alternative transcription start sites, promoter recognition sequences or alterations in the 5′ untranslated regions affecting folding or stability.

The level of RNA in a cell is usually controlled at the transcriptional and post-transcriptional levels. In bacteria, transcriptional regulation is commonly mediated via control of transcription initiation by RNA polymerase (RNAP) at the promoter [5]. Alternatively, post-transcriptional gene regulation is often mediated by the (often combined) action of non-coding or antisense RNA [6], RNA chaperones [7] and the activity of ribonucleases [8]. In the last two years, the use of high-throughput sequencing of cDNA (RNA-seq) has revealed that the complexity of the microbial transcriptome is much higher than previously predicted [9-13]. However, the high level of phylogenetic diversity within the bacterial kingdom has so far limited the possibilities for interspecies transcriptome comparison, since the species for which high resolution transcriptome maps are available are either too closely related (e.g. the *Enterobacteriaceae*) or too distantly related to allow meaningful comparisons at the evolutionary level.

The Epsilon-subdivision of the Proteobacteria (Epsilonproteobacteria) is a lineage which contains both pathogenic and non-pathogenic bacteria. The best studied examples of the former category are the human pathogens *Campylobacter jejuni* and *Helicobacter pylori*, which belong to the order *Campylobacterales*[14]. However, next to these important human pathogens, the Epsilonproteobacteria also contain chemolithoautotrophic microorganisms isolated from deep-sea vents [15,16], as well as the bovine rumen-colonising bacterium *Wolinella succinogenes*[17]. Despite the differences in ecological niches between the genera, and the genome sizes of Epsilonproteobacteria varying between 1.5 and 2.6 Mbp, genomic comparisons revealed that the Epsilonproteobacteria share similar transcription machinery including few sigma factors (with the notable exception of *Arcobacter butzleri*[18]), metabolic pathways and limitations, and have about half of the predicted genes in the genome in common with other Epsilonproteobacteria [14,15]. However, while these genomes share functionality, genome architecture and often low G + C content, the gene order and genome organisation have diverged significantly. This raises the question on how the genome and associated transcriptome copes with such large scale reorganisations of the genome when genera and species evolutionary diverge over time. To address this question, we have mapped the primary transcriptome of *C. jejuni* at the single nucleotide resolution using differential RNA-seq, have compared it with the primary transcriptome map of *H. pylori*[10] and have used genome sequences of 40 other taxa of the Epsilonproteobacteria to assess conservation and evolution of transcriptional circuitry in this highly variable group of bacteria.

## Results and discussion

### Differential RNA-seq analysis of the *C. jejuni* primary transcriptome

The *C. jejuni* NCTC 11168 genome contains 1643 annotated coding sequences (CDS), with only few stable RNA molecules known outside the ribosomal and transfer RNA species [19-21]. A single nucleotide resolution map of the *C. jejuni* transcriptome was generated by differential RNA-sequencing (dRNA-seq, [10]) using a motile variant of *C. jejuni* strain NCTC 11168 [22] and Roche 454 sequencing. To assess whether the dRNA-seq cDNA libraries are a good representation of transcribed sequences of *C. jejuni*, we compared the RPKM-values obtained for the CDSs from the non-enriched (−TEX) 454 cDNA sequencing with the previously published Illumina-based RNA-seq data for *C. jejuni* NCTC 11168 [21] and the signal intensity on a PCR-product based *C. jejuni* microarray [23,24] normalised to a genomic DNA reference [25]. There was a good correlation between the RPKM values for the two RNA-seq experiments and the microarray data (Additional file 1: Figure S1), with the best correlation observed between the two RNA-seq based approaches.

### Genome-wide identification of *C. jejuni* transcription start sites and promoters

**Figure 1:**
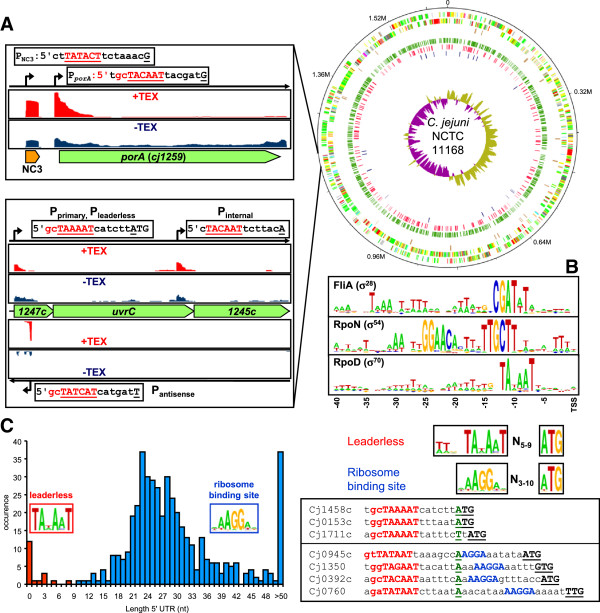
**Analysis of the primary transcriptome of *C. jejuni *NCTC 11168 by differential RNA-seq (dRNA-seq) analysis of transcription start sites (TSS). (A)** Genome map of *C. jejuni* strain NCTC 11168 with the outer three rings showing the annotated features of the plus and minus strand and annotated pseudogenes [19,20]. The inner rings show TSS associated with annotated features (green), antisense to annotated features (red), and non-coding RNAs (blue). The top inset shows the TSS and promoter of the *porA* gene and a putative non-coding RNA (NC3, CJnc140). The lower inset shows an operon with a primary TSS of a leaderless mRNA, an internal TSS and an antisense TSS. Red histograms (+TEX) show the cDNA read distribution in the cDNA library enriched for primary transcripts, dark blue histograms show cDNA read distribution in the non-enriched library (-TEX). **B)** WebLogo representations of the recognition sequences of σ^28^, σ^54^ and σ^70^, based on the sequences upstream of 26, 18 and 948 TSS, respectively (Additional file 5: Table S3). **C)** Identification of leaderless mRNAs of *C. jejuni* NCTC 11168. The graph shows the length distribution of 5′ untranslated regions (5′ UTRs) of 471 *C. jejuni* genes located downstream of a primary TSS. Red bars indicate proposed leaderless mRNAs, blue bars indicate a 5′ UTR with ribosome binding site (RBS). The insets show the motifs detected by MEME [28] upstream of the translational start codon of the *C. jejuni* genes with an RBS (right) and leaderless mRNAs (left). On the right side examples of 5′ UTRs are shown, with 3 proposed leaderless mRNAs, and 4 short 5′ UTRs containing a proposed RBS. The TSS (green, underlined), the σ^70^ -10 sequence (red) and the proposed RBS (blue) are highlighted. The full list of leaderless mRNAs is given in Additional file 13: Table S6.

The dRNA-seq data were subsequently used for the identification of transcription start sites (TSS) of primary RNAs, which are protected from digestion by Terminator Exonuclease (TEX) through their 5′-triphosphate modification [10,26]. The dRNA-seq method is based on the comparison of read distribution between the two cDNA-library enriched in primary 5′ ends (+TEX), and the non-enriched cDNA library (−TEX). Read distribution in the -TEX library displayed distribution throughout coding sequences, whereas treatment of RNA with Terminator Exonuclease for the + TEX library led to a typical cDNA read distribution resembling a sawtooth-like profile with an elevated 5′ flank [10,27] (Figure 1A, Additional file 2: Figure S2). TSS were annotated as primary, secondary, internal and antisense, based on their genomic location and association with annotated features, according to previously described criteria (Additional file 2: Figure S2) [10]. We identified a total of 992 TSS in the *C. jejuni* transcriptome (listed in Additional file 3: Table S1), which consisted of 510 primary and 11 secondary TSS located in intergenic regions which are associated with an annotated feature (CDS, pseudogene or stable RNA). A total of 12 genes are transcribed from two independent promoters (Additional file 4: Table S2), with 266 TSS located inside coding sequences or pseudogenes, and 206 TSS located antisense to coding sequences or annotated features.

Comparison of the dRNA-seq TSS with 53 previously published *C. jejuni* TSS, and 8 additional TSS determined by 5′ RACE analysis for this study (Additional file 5: Table S3), showed that 32/61 dRNA-seq TSS were identical, and an additional 17 were within 2 nt distance (81.8%, Additional file 6: Figure S3) of the previously described TSS, a difference which may be caused by the difficulty of 454 sequencing to accurately read long homopolymeric stretches [10]. In addition, due to the low number of TSS available for strain NCTC 11168, we used TSS from other reference strains and clinical isolates, and hence there may be strain differences in TSS as well [29]. The remaining 12 TSS were previously reported to lack recognisable promoter sequences, and as they were obtained by primer extension probably represent the 5′ end of processed RNA species rather than primary RNAs. This percentage match is similar as that described previously for *H. pylori*[10] and *Salmonella enterica* serovar Typhimurium [30,31]. Comparison with an independently performed study using Illumina sequencing published during preparation of this manuscript [29], showed that 795 TSS described in Additional file 3: Table S1 match TSS described in that study, but also highlights the identification of 197 additional TSS not described in [29]. There are several possible explanations for this discrepancy, which includes the different sequencing technology used (454 vs Illumina), as well as difference in growth conditions or growth phase of the *C. jejuni* cultures. It does highlight that the *C. jejuni* Supergenome described in [29] will undoubtedly be further expanded by future RNA-seq based studies with *C. jejuni*.

### Analysis of *C. jejuni* promoter sequences

*C. jejuni* has three sigma factors for promoter recognition, with σ^28^ and σ^54^ thought to be primarily involved in flagellar biogenesis, and σ^70^ to function as major vegetative sigma factor [20]. This was confirmed by dRNA-seq analysis, as only 26/992 (2.6%) of TSS were preceded by a putative σ^28^ recognition sequence (5′ CGATwt at 6–8 nt upstream of the TSS, Figure 1B) and 18/992 (1.8%) of TSS were preceded by a σ^54^ recognition sequence (5′ GGaa-N6-tTGCTt at 8–13 nt upstream of the TSS, Figure 1B) [32,33]. The remaining 948/992 (95.6%) of TSS were preceded by a gnTAnaAT motif at 4–8 nt upstream of the TSS (Figure 1B), consistent with a −10 Pribnow box for σ^70^[5]. As previously predicted [34], a −35 sequence was not present, but the sequences upstream of the −10 box showed a periodic signal centering on the −7, −17, −27 and −38 residues upstream of the TSS (Figure 1B, Additional file 7: Figure S4). We further analysed the average profile for 99 physico-chemical and geometrical DNA properties of the aligned σ^70^ promoters from position +1 to −51 (Additional file 7: Figure S4), including the corresponding 50 overlapping dinucleotides [35,36]. This highlighted the conservation of 16 dinucleotides individually and the overall nucleotide, dinucleotide and physical properties conservation in comparison (conservation measured by entropy). Furthermore, the overall nucleotide and dinucleotide conservation is quite similar, whereas some properties are partly higher conserved, especially the two measures slide and entropy at positions –27,–26. Slide is known to be indicative for DNA stiffness [37], which is related to the DNA entropy. This indicates that the right DNA stiffness at these positions might support promoter functioning. We also found a significant correlation of two physical properties (inclination, direction of deflection angle) of neighboured dinucleotides at positions –31/–32 –33/–34 (Additional file 7: Figure S4). Overall there was a good correlation between the nucleotide sequence and physico-chemical and geometrical DNA properties of the aligned σ^70^ promoters. In addition, there was no difference observed between σ^70^ promoters upstream of internal TSS and antisense TSS when compared to primary TSS and secondary TSS in intergenic regions (not shown).

### Genome-wide antisense transcription in *C. jejuni*

Within the 992 TSS, 206 were on the antisense strand of annotated features (antisense TSS, Additional file 3: Table S1, Additional file 8: Table S4), which confirmed the presence of genome-wide *cis*-antisense transcription, as recently described in other microbes [10,38-41]. We subsequently confirmed four antisense TSS by 5′ RACE (Additional file 5: Table S3), thus ensuring that the antisense TSS identified are not an artifact of the dRNA-seq technology. Antisense transcripts were often relatively short (27–285 nt in our dataset, average 114 nt), and many display a low number of reads, which may indicate spurious or pervasive transcription [41]. The presence of antisense TSS was not related to the level of transcription of the gene in either microarray or dRNA-seq, nor is antisense transcription related to specific functional categories of the genes opposite to the antisense TSS (Additional file 7: Table S4). Some genes have multiple antisense TSS, and antisense transcription was also detected opposite to transcriptionally active *C. jejuni* pseudogenes, which may allow for silencing of these pseudogenes via the activity of the double strand-specific ribonuclease III [29,42] or block the progress of RNA polymerase via transcriptional interference [43]. Antisense RNA may contribute to downregulation of parts of operons by post-transcriptional modification, without a requirement for transcriptional regulators. Alternatively, since some of the antisense TSS were located at the 3′ end of the coding sequence, they may function in transcript termination.

### Non-coding RNA

The annotations of the *C. jejuni* NCTC 11168 genome sequence [19,20] suggested the presence of several species of non-coding and stable RNAs, such as rRNAs, tRNAs, tmRNA, RNase P and the signal recognition particle (SRP) RNA. Furthermore, the presence of a thiamine pyrophosphate (TPP)-responsive riboswitch was predicted upstream of the *thiC* gene [19,21], as well as a possible purine riboswitch upstream of the *purD* gene [44], but no other ncRNA species were predicted or recognised, consistent with the absence of the Hfq RNA chaperone commonly associated with ncRNA-dependent regulation in bacteria [7,45]. A total of 29 putative non-coding and stable RNAs (ncRNAs) were identified in intergenic regions, scattered over the *C. jejuni* genome (Additional file 9: Table S5). We confirmed transcription of eight of these ncRNAs using Northern hybridisation (Additional file 10: Figure S5). Most of the ncRNAs detected were relatively short (30–100 nt), consistent with the relatively small and densily packed nature of the *C. jejuni* genome. Transcription of other predicted ncRNAs (tmRNA, RNase P and SRP RNA) was confirmed using dRNA-seq, with the SRP RNA also being detected by Northern hybridisation (Additional file 10: Figure S5). Comparison of the *C. jejuni* sRNAs recently described by Dugar *et al.*[29] and the earlier *C. jejuni* RNA-seq study by Chaudhuri *et al.*[21] showed a good correlation with the first study with 8 new ncRNAs described here, but only partial overlap with the second study, as two proposed non-coding RNAs matched (NC15/CJnc110 and NC8/CJnc190), with the rest either gene promoters (such as Intergenic_671549–671895 which encodes a *selW* ortholog [46]) or absent in our study.

The highest transcribed *C. jejuni* non-coding RNA (next to rRNA and tRNA) is located upstream of the *purD* (*cj1250*) gene, and a shorter version of this sequence was previously proposed as potential purine riboswitch [44]. However, the same region was recently identified in *H. pylori* to harbor a homolog of the abundant 6S RNA, a widespread regulator of RNA polymerase that was first described in *E. coli*[47]. Investigation of the 185 nt transcript (Additional file 11: Figure S6) showed that it started further upstream than the previously predicted purine riboswitch, and that it folds in a structure corresponding to that of bacterial 6S RNA [47,48], with a closing stem, central bubble and terminal loops (Additional file 11: Figure S6). The *E. coli* 6S RNA accumulates during exponential growth, and regulates the activity of σ^70^-containing RNAP by mimicking its open complex promoter structure, thus complexing σ^70^-cofactored RNAP [49]. In *E. coli*, RNAP releases itself from 6S RNA after a nutritional upshift by the production of a small product RNA (pRNA, 14–20 nt), originating in the central bubble [50]. Our original analysis did not show any such pRNA, but as our cut-off for cDNA reads was <18 nt, we also searched the <18 nt cDNA reads for sequences on the complementary strand of 6S RNA, and indeed found a 13 nt RNA antisense to the 6S RNA (Additional file 11: Figure S6) at a similar position as detected for one of the two pRNAs of *H. pylori* 6S RNA [10]. In *C. jejuni*, 6S RNA transcription is not significantly regulated in the different phases of exponential growth, and is not significantly altered upon growth cessation after exposure to pH 5.0 or 3.6 (Additional file 11: Figure S6), suggesting its role in *C. jejuni* may be distinct from that reported for *E. coli*.

### Leader peptides upstream of amino acid biosynthetic genes

For TSS that are >50 nt upstream of the annotated translation initiation codon (ATG, GTG or TTG), we searched the putative 5′ untranslated region (5′ UTR) for the presence of a small open reading frame (ORF) with a potential ribosome binding site (RBS, aAGGa) upstream, as was recently described for the *mfrX* gene upstream of the *C. jejuni mfrABE* genes [51]. Several small ORFs were thus identified and the length of the corresponding 5′ UTR was corrected. For three of these short ORFs, a functional prediction can be made based on their location upstream of the leucine, tryptophan and methionine amino acid biosynthetic operons in *C. jejuni* (Additional file 12: Figure S7) [52]. These three ORFs are likely to encode regulatory leader peptides, which couple transcription of amino acid biosynthetic genes to the availability of amino acid-coupled tRNAs [52], which has not been described for Epsilonproteobacteria. The small ORF (28 aa, tentatively named LeuL) upstream of the *leuABCD* (*cj1719c*-*1716c*) genes contains 5 Leu-codons at the C-terminal end of the polypeptide, which are all rare codons for leucine in *C. jejuni* (CUA, CUC and CUG), which together represent only 10.5% of the Leu codons in *C. jejuni*. Similarly, the short ORF (24 aa, tentatively named TrpL) upstream of the *trpEDFBA* (*cj0345*-*0349*) genes does contain a single Trp-codon at the C-terminal end of the polypeptide. Finally, a third short ORF (20 aa, tentatively named MetL) is located on a short RNA preceding the *metBA* (*cj1727c*-*1726c*) genes, with 3 Met-codons (Additional file 12: Figure S7). The RNAs encoding these ORFs all terminate shortly behind the stopcodon, and we propose that these polypeptides function as leader peptides, which allow transcription termination in the absence of ribosome stalling, and antitermination when the ribosome stalls due to the lack of availibility of tRNAs charged with the respective amino acid [52,53].

### 5′ untranslated regions and leaderless mRNAs

The average length of the 5′ untranslated regions (5′ UTRs) from 471 primary TSS ranged from 0 to 158 nt (average 30.6 ± 17.8 nt). A motif search using the MEME Motif discovery tool identified the sequence of 5′-aAGGa as conserved RBS motif (Figure 1C). The relatively short 5′ UTRs of the other promoters in intergenic regions are consistent with the *C. jejuni* genome being tightly packed, since >93% of the genome is thought to contain functional regions [20,54]. With the exception of the annotated TPP riboswitch upstream of the *cj0453* (*thiC*) gene [19], there were no metabolite-sensing riboswitches detected in the collection of 5′ UTRs. This is consistent with a previous study predicting an absence of these structures in *C. jejuni* and related bacteria [44].

Nineteen of the 5′ UTRs were <10 nt in length, with 12/19 of the TSS starting on the first nucleotide of the translation initiation codon, and these 5′ UTRs lacked a recognisable Shine-Dalgarno (ribosome binding site, RBS) sequence, with all the connected genes having an ATG startcodon (Figure 1C, Additional file 13: Table S6) and are preceded by a TAnAaT σ^70^ promoter sequence (Figure 1C, Additional file 13: Table S6). Such mRNAs are known as leaderless mRNAs [55], and were previously thought to be rare in bacteria. Leaderless mRNAs allow for translation during a range of physiological conditions, without competition for 30S ribosomes [55,56]. The genes translated from the *C. jejuni* leaderless mRNAs indeed encoded proteins predicted to be involved in stress-responses, like the DNA repair systems Nth endonuclease III (*cj0595c*) and MutY (*cj1620c*), the outer membrane efflux protein CmeD (*cj1031*) and the predicted multidrug efflux pump *cj1257c* (Additional file 13: Table S6).

### Comparison of primary transcriptomes of *C. jejuni* and *H. pylori*

The availability of dRNA-seq datasets for *H. pylori*[10] and *C. jejuni* (this study) allowed for a direct transcriptome comparison between two relatively distant species within the order Campylobacterales. Both species are pathogenic to man, have a similar genome size (~1.7 Mbp) and cellular morphology, and colonise mucus layers within the mammalian and avian gastrointestinal tracts.

### Gene order

**Figure 2:**
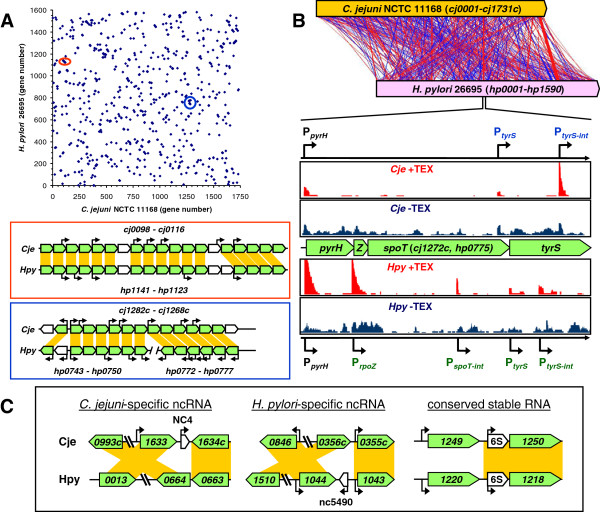
**The lack of genome synteny between *C. jejuni *and *H. pylori *at the level of gene order is reflected at the level of TSS/promoter location and non-coding RNAs. A)** A scatter plot of annotated gene numbers shows the lack of genome synteny and gene order between *C. jejuni* NCTC 11168 (Cje) and *H. pylori* 26695 (Hpy). The red and blue circles represent the two regions compared below the graph, with arrows representing conserved genes (green), genes only found in either *C. jejuni* or *H. pylori* (white). Orange bars indicate homologous genes. Line arrows above the gene representations indicate TSS and associated promoters. **B)** Alternative visualisation of genomic location of orthologs between *C. jejuni* NCTC 11168 and *H. pylori* 26695, based on pairwise BLASTP comparisons of annotated features, using the Artemis Comparison Tool (ACT) [60]. Red lines indicate the presence of an ortholog between *C. jejuni* and *H. pylori* in the same transcriptional orientation, whereas blue lines indicate inverted transcriptional orientation. The *spoT* genomic region [57,58] is used as example of a region with gene order conserved between the species, but a lack of conservation of transcriptional features. The dRNA-seq profiles of the *C. jejuni cj1274c*-*1271c* region and the *H. pylori hp0777*-*hp0774* region (+TEX = enriched for TSS, -TEX = not enriched) are shown above and below, respectively. Promoters associated with TSS are indicated in black (primary TSS), blue (internal TSS for *C. jejuni*) and green (internal TSS for *H. pylori*). **C)** Non-coding RNAs of *C. jejuni* and *H. pylori* are not conserved between these two species with the exception of the 6S RNA (right), and are associated with a lack of conservation of flanking genes. One example of each category (*C. jejuni*-specific ncRNA, *H. pylori*-specific ncRNA and a conserved stable RNA) is shown. Orange bars connect orthologous genes.

We first used BLASTP to compare the ORFs annotated in both genomes, and found they share 881 ORFs when only counting the highest scoring ortholog. These are however not ordered similary, as the gene order- based genome synteny was very low (Figure 2A, B), with only 10 regions where 5 or more orthologs were in the same order. The longest regions containing a conserved gene order are a ribosomal operon (*cj1708c-1688c*, *hp1320-1300*), the operon encoding a putative NADH-ubiquinone oxidoreductase (*cj1579c-1566c*, *hp1260-1274*) and the operon containing the F_1_F_0_ ATPase (*cj0098-0116*, *hp1141-1126*), but the latter two still contain insertions of non-orthologous genes within the region. Other regions, such as the region containing the *spoT* gene [57,58] are contiguous in the *C. jejuni* genome, but split over two regions in the *H. pylori* genome [59] (Figure 2A). We used one of these conserved regions (*cj1274c-1271c*, *hp0777-0774*) to directly compare the primary transcriptomes of both organisms (Figure 2B). Even though the gene order is conserved, the location of promoters is not. Both sets of genes are transcribed from a σ^70^-dependent promoter upstream of the *pyrH* gene, but the location of the internal promoters differs completely between *C. jejuni* and *H. pylori*, with the latter having an additional promoter upstream of *rpoZ*, and although both genomes have an internal promoter in the *tyrS* gene, its location is not conserved (Figure 2B).

### Non-coding RNA

Similarly, non-coding RNAs are not conserved between *C. jejuni* and *H. pylori*[29], with the exception of the stable RNAs like the 6S RNA. When the genomic locations of one *H. pylori* ncRNA (nc5490 [10]) and one *C. jejuni* ncRNA (NC4, CJnc170) were compared (Figure 2C), this showed that the neighbouring genes are not conserved between the species, which may explain the species-specificity of the ncRNAs. One possible explanation for the uniqueness of the ncRNAs identified here, may be that these ncRNAs are generated and deleted during genome reorganisations and gene reshuffling. The exception is the 6S RNA, which is in both genomes upstream of the *purD* gene (Figure 2C), although the upstream gene differs, and also there is significant sequence difference between the 6S RNA genes of both organisms [10].

### Antisense transcripts and internal promoters

**Figure 3:**
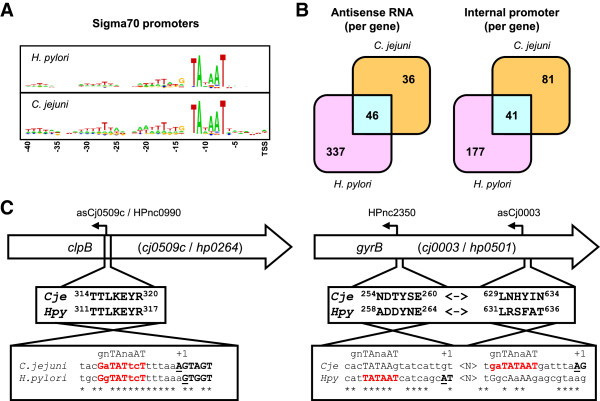
**Lack of conservation of antisense promoters and internal promoters between *C. jejuni *and *H. pylori*. A)** Comparison of the σ^70^ recognition sequences of *C. jejuni* and *H. pylori*, demonstrating that there is little difference between σ^70^ recognition sequences in the two species, with the σ^70^ -10 sequence of both species being gnTAnaAT. **B)** Comparison of the number of genes with antisense TSS and internal TSS in *C. jejuni* and *H. pylori*. Both species share 46 orthologs displaying antisense transcription in both species, whereas 41 shared orthologs have an internal promoter in both species. The lower number of antisense and internal promoters in *C. jejuni* is due to the lower coverage of 454 sequencing-based dRNA-seq. **C)** Examples of conservation and lack of conservation of antisense transcription in *C. jejuni* and *H. pylori*. Of the 46 genes with an antisense TSS shared between *C. jejuni* and *H. pylori*, only 2 out of 46 have the antisense TSS in the same position. This is shown for the *clpB* (*cj0509c* / *hp0264*) gene. The other 44 genes have the antisense promoters in different locations in the gene, as is shown for the *gyrB* (*cj0003* / *hp0501*) gene. The boxes below the genes show the protein motifs encoded opposite to the antisense promoters, while the boxes below the protein sequence compare the antisense promoters. Asterisks indicate conserved residues, black and bold typeface indicates the antisense RNA sequence, the underlined residue is the transcript start site (TSS, +1), while red and bold typeface indicates the -10 sequence of the σ^70^ promoter. Complete information on antisense and internal promoters can be found in Additional file 19: Figure S9 and Additional file 18: Table S10 and Additional file 20: Table S11.

Both the *C. jejuni* and *H. pylori* genomes only encode σ^28^, σ^54^ and σ^70^ sigma factors, with the large majority (>95%) of promoters being transcribed by σ^70^[10]. The *H. pylori* and *C. jejuni* σ^70^ promoters show a high degree of homology, both with the gnTAnaAT motif as −10 box [10], and higher conservation of the T-residues at −7, −17, −27 and −38 (Figure 3A). To compare for conservation of the transcriptional landscape between *C. jejuni* and *H. pylori*, we compared the genes in both genomes which have a) antisense transcription or an internal promoter and b) an ortholog in the comparator genome. This allowed for the comparison of 383 *H. pylori* genes with antisense transcription detected [10] with 82 *C. jejuni* genes for which antisense transcription was detected. Of these only 46 of these genes displayed antisense transcription in both *C. jejuni* and *H. pylori* (Figure 3B), however, for only two of these genes (*cj0509*/*hp0264* and *cj0774*/*hp1576*) the location of the antisense TSS was conserved. The antisense RNA in the *clpB* (*cj0509c*/*hp0264*) gene is located in a part encoding a conserved sequence in the ClpB protein, with the antisense RNA being of identical length (105 nt), with both promoter and asRNAs being ~80% identical in DNA sequence. In contrast, 44/46 other asRNAs were located in different parts (albeit sometimes closely located) of the corresponding *C. jejuni* and *H. pylori* genes, as is shown in Figure 3C for the *gyrB* (*cj0003*/*hp0501*) gene, with the *H. pylori* asRNA being located in the first half of the gene, and the *C. jejuni* asRNA being located in the part encoding the C-terminal end of the protein. The σ^70^ promoter motifs were not conserved in these two genomes, with in both cases the highly conserved −7 T residue being altered, thus probably inactivating the σ^70^ recognition sequence. Similar results were obtained with the 218 internal promoters of *H. pylori* and 122 internal promoters of *C. jejuni*. Only 41 of these internal promoters were located in orthologs shared between *H. pylori* and *C. jejuni* (Figure 3B), and of these 41, only six have the internal promoter in the same position in the gene, thus highlighting the lack of conservation of the transcriptional landscapes between *H. pylori* and *C. jejuni*.

### Predictive comparisons with other Epsilonproteobacterial genomes

**Figure 4:**
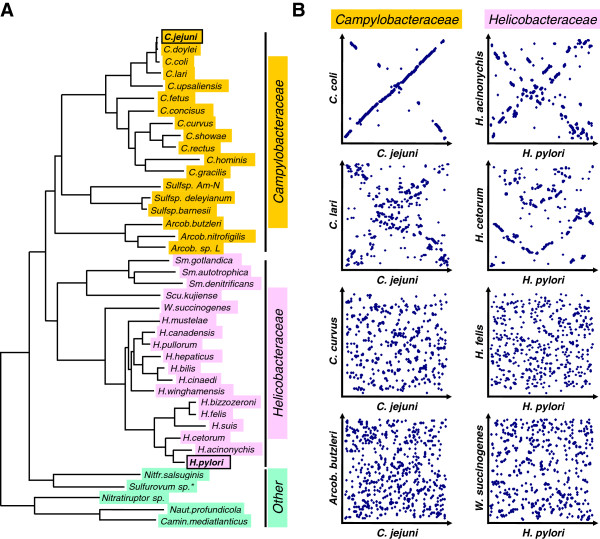
**Comparative genomics of the Epsilonproteobacteria highlights a lack of gene order-based genome synteny throughout this lineage. A)** Phylogenetic tree of 42 members of the Epsilonproteobacteria, based on comparison of 16S rDNA sequences. Members of the *Campylobacteraceae* (the genera *Campylobacter*, *Sulfurospirillum* (*Sulfsp*) and *Arcobacter* (*Arcob*)) are highlighted in orange, members of the *Helicobacteraceae* are highlighted in pink (the genera *Helicobacter*, *Wolinella*, *Sulfurimonas* (*Sm*) and *Sulfuricurvum* (*Scu*)), and other Epsilonproteobacteria are highlighted in green (the genera *Nitratifractor* (*Nitfr*), *Sulfurovum*, *Nitratiruptor*, *Caminibacter* (*Camin*) and *Nautilia* (*Naut*)). *Sulfurovum* sp. AR is not included in this tree as a full 16S rDNA sequence was not available from the genome sequence. **B)** Selected scatter plots of gene order-based genome synteny comparisons of *C. jejuni* NCTC 11168 with other members of the *Campylobacteraceae*, and *H. pylori* 26695 with other members of the *Helicobacteraceae*. The full comparisons are shown in Additional file 15: Figure S8, full information is given in Additional file 16: Table S8. Both the *Campylobacteraceae* and *Helicobacteraceae* show a rapid loss of genome synteny, even within the species *Campylobacter* and *Helicobacter*.

To investigate whether the lack of conservation of transcriptional landscapes between *C. jejuni* and *H. pylori* were dependent on phylogenetic differences, we used comparative genomics and genome synteny analyses with the genomes of 40 other Epsilonproteobacteria (Additional file 14: Table S7). These included 18 members of the *Campylobacteraceae* (12 *Campylobacter* spp, 3 *Arcobacter* spp and 3 *Sulfurospirillum* spp), 18 members of the *Helicobacteraceae* (13 *Helicobacter* spp, 3 *Sulfuromonas* spp, *Sulfuricurvum kujinense* and *W. succinogenes*), and 6 species of other Epsilonproteobacteria found in deep-sea hydrothermal vents. A phylogenetic tree based on 16S rDNA sequences (Figure 4A) shows the phylogenetic relationships between the investigated species, and shows the subdivision of *Campylobacter* spp into thermophilic (*C. jejuni* to *C. upsaliensis*) and non-thermophilic species (other *Campylobacter* spp), and within the *Helicobacter* spp the subdivision into gastric *Helicobacter* spp (*H. pylori* to *H. bizzozeroni*) and enterohepatic *Helicobacter* spp (other *Helicobacter* spp with the exception of *H. mustelae*).

### Gene order

We subsequently used the annotated features to compare genome synteny of all these species with *C. jejuni* NCTC 11168 and *H. pylori* 26695 (Additional file 15: Figure S8, Additional file 16: Table S8). From these analyses it is clear that gene order -based genome synteny is only conserved between closely related species (i.e. *C. doylei* and *C. coli* for *C. jejuni*, and *H. acinonychis* and *H. cetorum* for *H. pylori*), with genome synteny rapidly lost beyond these closely related species (Figure 4B). This progressive lack of genome synteny may explain the large differences between the experimentally determined *C. jejuni* and *H. pylori* transcriptomes, and may also explain the lack of conservation of non-coding RNAs within this phylogenetic clade (Figure 2C) [29].

### Leaderless mRNA

**Figure 5:**
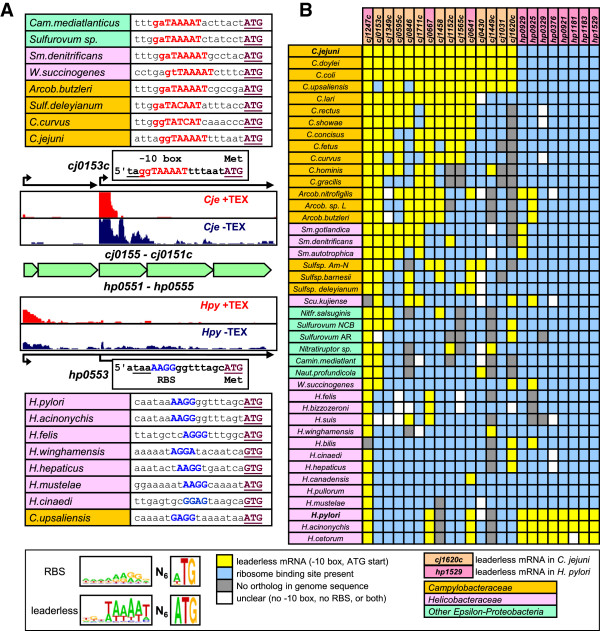
**Prediction of leaderless mRNAs in the Epsilonproteobacteria suggests evolutionary conversion to and from leaderless mRNAs. A)** The presence or absence of a leaderless mRNA can result in fusion or separation of cistrons, as shown for the leaderless *cj0153c* gene and the leadered *H. pylori hp0553* ortholog. The dRNA-seq profiles for the *cj0155c*-*cj0151c* and *hp0551*-*hp0555* genes show that *cj0153c* gene has a TSS with a σ^70^ -10 sequence, whereas *hp0551*-*hp0555* is transcribed as a single multicistronic mRNA, and *hp0553* has a potential ribosome binding site (RBS). The translational start site (ATG, GTG or TTG, brown), the proposed σ^70^ -10 sequence (red), and the proposed RBS (blue) are shown for examples from *Campylobacteraceae* (orange), *Helicobacteraceae* (pink) and other Epsilonproteobacteria (green). **B)** Prediction of leaderless mRNAs in the Epsilonproteobacteria, based on the sequences directly upstream of the translational start codon of 23 genes which are leaderless mRNAs in either or both *C. jejuni* NCTC 11168 and *H. pylori* 26695. The 5' UTRs were scored for the presence of gnTAnaAT or AG-rich RBS-like sequences, as shown in Figures 1C and 5A. The full set of annotated sequences and gene numbers is available in Additional file 17: Table S9. Predictions are colour-coded: yellow for of a leaderless mRNA, blue for a ribosome binding site, grey for the absence of an ortholog, and white when neither a RBS nor a suitable -10 sequence was detected. Members of the *Campylobacteraceae* are indicated in orange, members of the *Helicobacteraceae* in pink, and other Epsilonproteobacteria in green, and are ordered by the number of predicted leaderless mRNAs they have in common with *C. jejuni*. The genes are given at the top, with orange representing *C. jejuni*, pink *H. pylori*. A WebLogo representation of the upstream sequences and startcodons is shown for leaderless mRNAs and RBS-containing 5′ UTRs.

We also assessed the difference between Epsilonproteobacteria at the gene level, using the difference between leaderless and leadered mRNAs (Figure 1C). An initial comparison of leaderless mRNAs between *C. jejuni* and *H. pylori* showed clear differences, as only 3/23 genes are leaderless in both organisms, with 12 leaderless in *C. jejuni* only, and 8 leaderless in *H. pylori* only. One example of such difference is shown in Figure 5A, for the *C. jejuni cj0153c* gene. This gene is located in a conserved five-gene region (*cj0155c-0151c* and *hp0551-0555*), but is leaderless in *C. jejuni*, effectively splitting the five genes into two separate operons, while there is only a single five-gene operon in *H. pylori* (Figure 5A). The intergenic regions showed clear differences with the σ^70^ -10 sequence (ggTAAAAT) in *C. jejuni* and an RBS in *H. pylori* (AAGGG). As the *cj0155c-0151c* genes are conserved throughout the Epsilonproteobacteria, we compared the intergenic regions between the *cj0154c* and *cj0153c* orthologs in all 42 genomes, and this showed that the majority of genomes (27/42) contain a predicted σ^70^ -10 sequence, while 13/42 contain a recognisable RBS sequence, with 2 genomes containing neither (Additional file 17: Table S9). The RBS was only present in *Helicobacter* spp, and interestingly also in *C. upsaliensis*, which is surprising in view of its close phylogenetic relationship to *C. jejuni*, and suggests a secondary, independent evolutionary change in *C. upsaliensis* or the effect of a natural transformation event (Figure 5A).

We expanded this search to all 23 genes which are leaderless in either *C. jejuni* or *H. pylori* in all 42 Epsilonproteobacterial genomes. All genomes were searched for orthologs of the *C. jejuni* and *H. pylori* genes, and the −30 to +3 sequences (counted until the translational startcodon) were searched manually for σ^70^ -10 box and ATG startcodon (gnTAnaAT-N_5-9_-ATG) [61] and potential ribosome binding site and all three possible startcodons (aAGGa-N_3-10_-aTG), and were also used for a MEME motif search (Figure 5B, Additional file 18: Table S10). From the overview presented in Figure 5B, it is clear that the predicted leaderless or leadered mRNAs do not strictly follow the 16S rDNA-based phylogenetic tree (Figure 4A). While *C. jejuni*, *C. doylei* (a subspecies of *C. jejuni*) and *C. coli* cannot be distinguished in this analysis, this is also mostly true for *H. pylori*, *H. acinonychis* and *H. cetorum*, mirroring the genome synteny analyses (Figure 4B, Additional file 15: Figure S8). Only a single gene (*cj1247c* in *C. jejuni*, *hp0820* in *H. pylori*) is a predicted leaderless mRNA in all species containing this gene, which is always upstream of the *uvrC* DNA repair gene, again supporting an important role of leaderless mRNAs in stress responses [55]. Interestingly, the *Sulfurimonas* spp (members of the *Helicobacteraceae*) have more leaderless mRNAs in common with the *Campylobacter* spp than with other members of the *Helicobacteraceae*, while there is virtually no conservation of leaderless mRNAs within the genus *Helicobacter* (Figure 5B). Another surprise was that the enterohepatic *Helicobacter* spp had only one or two predicted leaderless mRNAs in common with other Epsilonproteobacteria, which suggests that they may contain a completely different set of leaderless mRNAs, something which was not followed up for this study.

### Antisense transcription and internal promoters

Next we compared five internal and three antisense promoters conserved between *C. jejuni* and *H. pylori* (Figure 3B) in the other Epsilonproteobacterial genomes. BLAST-searches were used to identify the corresponding regions in the respective orthologs, and sequences were searched for potential σ^70^ -10 box both manually and using MEME (Additional file 19: Figure S9, Additional file 18: Table S10, Additional file 20: Table S11). As with the leaderless mRNAs, there was no full conservation of internal or antisense promoters, although for the promoter internal to *cj0705* this can be linked to the genomic organisation: in *W. succinogenes* the *cj0705* ortholog is fused to the downstream *cj0706* ortholog (thus not requiring a promoter), whereas in *Caminibacter mediatlanticus* and *Nautilia profundicola* the downstream *cj0706* ortholog is absent (Additional file 19: Figure S9, Additional file 18: Table S10). Similarly, there is a good albeit imperfect correlation between the presence of the *cj0100* and *cj0101* (*parAB*) orthologs and the presence of an internal promoter in *cj0099* (Additional file 19: Figure S9, Additional file 18: Table S10). With regard to the antisense promoters, most of the *cj0509c* (*clpB*) orthologs in the Epsilonproteobacteria contain a predicted σ^70^ -10 box at the equivalent position (35/42, Additional file 20: Table S11), whereas the predicted antisense promoters in the *cj0003* (*gyrB*) orthologs are confined to the phylogenetically closely related species, i.e. the *C. jejuni*, *C. doylei* and *C. coli* group vs the *H. pylori*, *H. acinonychis* and *H. cetorum* group, consistent with evolutionary relationships between these species (Figure 4A, Additional file 19: Figure S9, Additional file 20: Table S11).

### Leader peptides

Finally, we also searched the Epsilonproteobacterial genomes for orthologs of the leucine, tryptophan and methionine amino acid biosynthetic genes, and whether they contained potential leader peptides upstream (Additional file 12: Figure S7, Additional file 21: Table S12). While LeuL orthologs (20–35 aa peptide with several leucines at the C-terminus) were found in most Epsilonproteobacterial genomes with leucine biosynthetic genes, TrpL and MetL orthologs were only found in *C. jejuni*, *C. coli* and *C. doylei*, and were absent from other Epsilonproteobacteria with tryptophan or methionine biosynthetic genes (Additional file 12: Figure S7, Additional file 21: Table S12). As with the previous examples, this suggests that there are clear differences between genomes and transcriptomes within the Epsilonproteobacteria, and also that changes in genome content and gene order have necessitated the development of differential forms of transcriptional, post-transcriptional and possibly translational regulation of gene and protein expression.

## Conclusions

In this study, we present the primary transcriptome of *Campylobacter jejuni* at single nucleotide resolution, obtained by using differential RNA-sequencing analysis using 454 sequencing. Our analysis confirms that the original analyses of the *C. jejuni* genome [19,20,34] have indeed underestimated its versatility and complexity, with a wealth of non-coding and antisense RNAs, as well as intragenic promoters and leaderless mRNAs. All these features are likely to contribute to the success of *C. jejuni* as pathogen, allowing it to survive in the food chain and infect different hosts. Our analysis complements and supplements the previously released and reannotated genome sequence and protein interactome maps for *C. jejuni*[19,20,62], and RNA-seq analyses using Illumina sequencing [21,29]. The large number of transcription start sites found in the relatively small *C. jejuni* genome supports the findings in other bacteria, where a much larger number of TSS have been detected than was expected, e.g. the >17,000 TSS identified in *Sinorhizobium meliloti*[63], which has a much larger genome, megaplasmids and multiple sigma factors when compared to the Epsilonproteobacteria.

The availability of the dRNA-seq datasets for two related members of the Epsilonproteobacteria has allowed for the first high resolution comparison of primary transcriptomes at the single-nucleotide level of related, but independent species (*C. jejuni* and *H. pylori*). All characterised members of the Epsilonproteobacteria have relatively small genomes (1.5 - 3 Mbp), and show high levels of variation, probably due to a relative scarcity of DNA repair mechanisms and the exchange of DNA by natural transformation and horizontal gene transfer [19,64,65]. Interestingly, despite the variability in both the genome and transcriptomes of these organisms, there were parts which showed high levels of conservation (like operons encoding ribosomal proteins) and others which showed no conservation at all (Additional file 15: Figure S8). With regard to transcriptome organisation, there were the large scale differences already predicted by the comparison of genome sequences (Additional file 15: Figure S8) [14,66], but also very subtle differences with respect to coupling and uncoupling of transcriptional networks, for instance by the appearance and disappearance of promoters coupled to leaderless mRNAs (Figure 5), and generation and absence of internal and antisense promoters (Additional file 19: Figure S9).

Overall, there was very low synteny between the regulatory features of the *C. jejuni* and *H. pylori* transcriptomes with respect to the position and sequence of internal promoters, antisense RNAs and non-coding RNAs, with the exception of the ancestral 6S RNA. Orthologous sequences to the *C. jejuni* ncRNAs and asRNAs were only found in other *C. jejuni* strains and partially in closely related species (*C. doylei*, *C. coli*), and similarly conservation of *H. pylori* features was limited to other *H. pylori* strains, and partially with *H. acinonychis* and *H. cetorum*[29,67]. All this suggests that many of these regulatory features of the transcriptomes of Epsilonproteobacteria will have developed after genera have evolutionary split from a common ancestor, and are likely to be in constant flux depending on their ecological niches and its influence on genome reorganisation, mutation frequency and horizontal gene transfer. The large differences observed between *C. jejuni* and *H. pylori*, and even the differences observed between *C. jejuni* strains [29] promises that future RNA-seq experiments with other Epsilonproteobacteria can be expected to show up many new and exciting features.

## Methods

### Bacterial strains and growth conditions

A motile variant of *C. jejuni* strain NCTC 11168 [20] was used throughout this study, and cultured in a MACS-MG-1000 controlled atmosphere cabinet (Don Whitley Scientific) under microaerobic conditions (85% N_2_, 5% O_2_, 10% CO_2_) at 37°C. For growth on plates, strains were grown on blood plates (Blood Agar Base 2 (BAB), 1% yeast extract, 5% horse blood (Oxoid) with Skirrow supplements (10 μg ml^-1^ vancomycin, 5 μg ml^-1^ trimethoprim, 2.5 IU polymyxin-B). Broth culture was carried out in Brucella broth (Becton, Dickinson & Company) [22].

### RNA preparation, cDNA library construction and Roche 454 pyrosequencing

RNA was isolated from the motile *C. jejuni* strain NCTC 11168 [22], grown to late log phase (OD_600_ = 0.21). Total RNA was purified omitting size selection, to avoid the loss of small RNA molecules. The exclusion of rRNA and tRNA was also omitted, to avoid the potential loss of other RNA species. RNA was isolated using hot phenol [68], to ensure that small RNAs would not be removed by the extraction procedure. The RNA was treated with DNase I to remove residual genomic DNA, followed by optional treatment with Terminator Exonuclease (TEX, Peicentre Biotechnology) for enrichment of primary RNAs [10,26], and treatment with Tobacco Acid Phosphatase (TAP, Cambio, UK) to generate 5′-P ends for downstream ligation of 454 adapters [10]. After ligation of an RNA oligonucleotide to the phosphorylated 5′-ends of RNA, and polyadenylation of RNA, first strand cDNA was generated using an oligo-dT containing 454-B primer (Additional file 22: Table S13). The cDNA fragments were barcoded and amplified, and used for generation of cDNA libraries for the 454 FLX system at Vertis Biotech, Germany. These libraries were subsequently analysed using a Roche FLX sequencer located at Liverpool University, UK, as previously described [10]. The enrichment procedure significantly reduced the level of 23S and 16S rRNA, but led to an increase in 5S rRNA, while tRNA levels were not altered [10].

### Mapping of 454 reads and annotation of transcription start sites

Sequencing reads were grouped based on the barcode tag, the 5′ adapter was clipped, and reads of >70% A were removed. The remaining reads were aligned against the *C. jejuni* genome NCTC 11168 genome sequence using Segemehl version 0.0.9.3 [69], and converted into number of reads per nucleotide position. Graphs representing the number of mapped reads per nucleotide were visualized using the Integrated Genome Browser software from Affymetrix [10,70]. TSS were manually annotated based on a higher and characteristic cDNA coverage of the 5′-end of a given cDNA in the library constructed with terminator exonuclease-treated RNA. Genomes were annotated and analysed using Artemis [60]. Transcript levels of individual genes were expressed as Reads Per Kilobase per Million mapped reads (RPKM) values, calculated after mapping of reads using CLC Genomics Workbench v5 (CLC Bio). Thermodynamical and geometrical dinucleotide properties of DNA sequences were visualised using the DiProDB browser [36], whereas sequence conservation was visualised using the WebLogo program [71]. Sequence alignment was performed using ClustalX2 [72], phylogenetic analyses with Phylip v3.69 [73], and sequence motif searches were done using the MEME suite [28].

### Comparative genomics

The complete genome sequences or contigs of 42 species of the Epsilonproteobacteria (Additional file 16: Table S8) were downloaded from the NCBI Genomes database (http://www.ncbi.nlm.nih.gov/genome) or via the PATRIC website at the Virginia Tech University (http://patricbrc.vbi.vt.edu/) [74]. Incomplete genome sequences were concatenated into a single genome sequence using the Union program of the mEMBOSS suite [75] in the order of the contigs provided. Pairwise comparisons of annotated features were made using BLASTP [76], with a E-value threshold of 0.000001, and sorted to record the highest match with the annotated features of the *C. jejuni* NCTC 11168 or *H. pylori* 26695 genome. The respective gene numbers were extracted and used as X,Y coordinates in a scatterplot, essentially as described for the GeneOrder 4.0 program [77]. To identify orthologs of genes with leaderless mRNAs, internal promoters or antisense RNAs in *C. jejuni* NCTC 11168 and *H. pylori* 26695, the annotated features and genomic DNA sequence were probed with BLASTP and TBLASTN with the BioEdit program (http://www.mbio.ncsu.edu/bioedit/bioedit.html). BLASTP alignments were used to identify corresponding regions in the genes for analysis of promoter conservation based on the -10 sequence both manually (for 5′-gnTAnaAT sequences) and with MEME motif searches.

### Northern blot analysis

RNA was separated on 6% Tris-borate-EDTA polyacrylamide (PAA) gels, containing 8.3 M urea. Each lane contained 10 μg of total RNA, isolated from *C. jejuni* NCTC 11168 grown to early, mid and late logarithmic phase, or subjected to 30 min incubation in Brucella broth of pH 5.0 or pH 3.6. After separation, RNA was transferred onto HybondXL membranes (GE Healthcare) by electroblotting and cross-linked to the membrane. Membranes were prehybridized in Rapid-hyb buffer (GE Healthcare) at 42°C, followed by hybridization with 10 pmol [γ-32P]-ATP end-labeled oligodeoxynucleotides (Additional file 22: Table S13) for 1 h. After washing 3 times for 15 min in 5×, 1×, and 0.5× SSC–0.1% SDS solutions (42°C), signals were visualized on a phosphorimager (FLA-5000 Series, Fuji) [10].

### 5′ RACE

RNA adapter (Additional file 22: Table S13) was ligated to the 5′ end of both TAP-treated and untreated RNA. 5′ RACE was performed as described previously [51,78]. First-strand cDNA synthesis was performed using 2 pmol Random hexamer (GE Healthcare, USA) and Thermoscript RT (Invitrogen) according to manufacturer’s instructions. The RNA template was removed at the end by incubating the samples for 20 minutes at 37°C in the presence of 5 units RNase H (New England Biolabs, Ipswich, USA). PCR amplification was performed using gene-specific primers (Additional file 22: Table S13) and a 5′ adapter-specific DNA primer (Additional file 22: Table S13). The resulting PCR products were cloned into the pGEM-T_easy_ cloning vector (Promega, Leiden, The Netherlands) and the nucleotide sequence of the inserts was determined.

## Availability of supporting data

The dRNA-seq histogram files and associated information have been deposited in the GEO database with accession number GSE49312 (http://www.ncbi.nlm.nih.gov/geo/query/acc.cgi?acc=GSE49312). The raw sequencing data have been uploaded as 454 SFF files into the Short Read Archive with accession number SRX326863 (http://www.ncbi.nlm.nih.gov/sra/?term=SRX326863). An annotated Artemis entry has been created which contains the information of Additional file 3: Table S1 for use with the *C. jejuni* NCTC 11168 genome sequence (Accession number NC_002163) and is included with the article as Additional file 23.

## Abbreviations

cDNA: Copy DNA; TSS: Transcription start site(s); RNAP: RNA polymerase; dRNA-seq: Differential RNA-sequencing; TEX: Terminator exonuclease; TAP: Tobacco acid phosphatase; Mbp: Million base pairs; nt: Nucleotide(s); RACE: Rapid amplification of cDNA ends; RPKM: Reads per kilobase per million mapped reads; tmRNA: Transfer-messenger RNA; RNase: Ribonuclease; SRP: Signal recognition particle; TPP: Thiamine pyrophosphate; ncRNA: Non-coding RNA; sRNA: Small RNA; pRNA: Product RNA; 5’ UTR: 5’ untranslated region; RBS: Ribosome binding site; ORF: Open reading frame; BAB: Blood agar base 2.

## Competing interests

The authors declare that they have no competing interests.

## Authors’ contributions

IP and AHMvV designed the research; IP, MR and BMP performed the experimental research and analysed data; AHMvV, MR and TW performed the bioinformatic analyses, AHMvV wrote the paper, on which all authors commented. All authors read and approved the final manuscript.

## Supplementary Material

Additional file 1: Figure S1 Comparison of expression levels of *C. jejuni* genes on RNA-seq and microarray, using the Illumina-based quantitative RNA-seq data from Chaudhuri et al. [21], with and microarray and differential RNA-seq data (this study). A) Comparison of RNA-seq and the -TEX reads from differential RNA-seq analysis, based on log_2_(RPKM + 1) values [21] for 1509 genes. B) Comparison of RNA-seq [21] and normalised microarray expression levels for 1542 genes. C) Comparison of differential RNA-seq and normalised microarray expression levels for 41423 genes. Click here for file

Additional file 2: Figure S2 Identification of transcription start sites in *C. jejuni*. A) Schematic representation of the different types of transcription start sites, with primary and secondary TSS being located at ≤ 500 nt from the translational startcodon of the respective gene. TSS can have multiple associations, as shown for the primary and internal TSS within the first gene. B) Venn diagram representing the overlap between the different classes of TSS identified for *C. jejuni*. C) The *cj1316c* is transcribed from both a primary and secondary TSS (left) whereas the *ftsZ* (*cj0696*) gene is transcribed from an internal promoter located in the coding sequence of the upstream *ftsA* (*cj0695*) gene (right), allowing intraoperonic differentiation of transcript levels. Translational start codons and putative RBS are underlined, TSS are shown underlined in bold typeface, and extended and normal −10 sequences for σ^70^ and σ^28^ are indicated in bold typeface. Click here for file

Additional file 3: Table S1 *C. jejuni* transcription start sites and annotated promoter sequences identified by dRNA-seq. Click here for file

Additional file 4: Table S2 *C. jejuni* genes with primary and secondary promoters. Click here for file

Additional file 5: Table S3 Comparison of *C. jejuni* TSS identified by dRNA-seq with TSS identified by 5′ RACE and primer extension. Click here for file

Additional file 6: Figure S3 Comparison of *C. jejuni* TSS identified by dRNA-seq with those previously published (Additional file 5: Table S3). The histogram indicates the number of distances between 61 TSS identified in *C. jejuni* by primer extension and 5′ RACE with those determined by dRNA-seq. Click here for file

Additional file 7: Figure S4 Sequence conservation in *C. jejuni* σ^70^ promoters is matched by conservation in physico-chemical properties. A) WebLogo representation of the −50 to +1 sequences of σ^70^ promoters in *C. jejuni* (Additional file 3: Table S1). B) Profiles for 99 physical DNA properties taken from DiProDB [36] (upper panel), conservation of dinucleotides (panel 2) and conservation of 99 physical properties [panel 3; for comparison: red curve nucleotide conservation (corresponding to height of weblogo), green dinucleotide conservation]. The two curves peaking at −26, −27 are slide and entropy. The lower panel shows the significance of correlation of physical properties of neighboured dinucleotides (uncorrected p-values). The two curves peaking at −18 are inclination and direction of the deflection angle. Click here for file

Additional file 8: Table S4 Characteristics of cis-antisense RNAs of *C. jejuni*. Click here for file

Additional file 9: Table S5 *C. jejuni* non-coding RNAs in intergenic regions. Click here for file

Additional file 10: Figure S5 Identification of non-coding RNAs (ncRNAs) in the *C. jejuni* transcriptome and independent confirmation of their transcription using Northern hybridisation. For 7 ncRNAs, the dRNA-seq histograms are shown, with the red histograms representing the + TEX cDNA library enriched for primary transcripts, and the blue histograms representing the non-enriched -TEX cDNA library. Genes/ncRNAs are shown above the histograms with the arrows representing their transcriptional direction, while small arrows indicate the position of transcription start sites and orientation of promoters. Below the histograms, Northern hybridisations are shown with independent RNA-samples, isolated in early, mid and late log growth phases, and after 30 minutes exposure to pH 5.0 and pH 3.6. Relevant marker sizes are indicated on the right hand side. The scissor symbol above the CjNC8 ncRNA indicates a putative post-transcriptional modification site, resulting in a mature RNA of 70 nt. The SRP RNA is included as control. Full information on the ncRNAs can be found in Additional file 9: Table S5. Click here for file

Additional file 11: Figure S6 Identification and characterisation of the *C. jejuni* 6S RNA. (A) The 6S RNA is encoded directly upstream of the *purD* (*cj1250*) gene, and is transcribed on the leading strand from a σ^70^ promoter upstream of the TSS (shown above the histograms). The product RNA (pRNA) transcribed from the complimentary strand is shown below. (B) Predicted folding of the *C. jejuni* 6S RNA. The sequence used to transcribe the complementary pRNA is marked by a green box. (C) Transcription of the 185 nt 6S RNA is constitutive during exponential growth and during acid shock, as demonstrated using Northern hybridisation. Click here for file

Additional file 12: Figure S7 Identification of *C. jejuni* putative leader peptides allowing coupling of amino acid availability to downstream expression of the amino acid biosynthetic pathways for tryptophan, methionine and leucine. The sequence of the leader peptide is indicated, with the corresponding regulatory amino acid in red typeface. When similar leader peptides are predicted to be present in other Epsilonproteobacteria (based on location and presence of a ribosome binding site), their sequence is included. The dRNA-seq histograms for *C. jejuni* are shown, with the red histograms representing the + TEX cDNA library enriched for primary transcripts, and the blue histograms representing the non-enriched -TEX cDNA library. Click here for file

Additional file 13: Table S6 Leaderless mRNAs of *C. jejuni* NCTC 11168. Click here for file

Additional file 14: Table S7 Epsilonproteobacterial and other genome sequences used in this study. Click here for file

Additional file 15: Figure S8 Lack of gene order-based genome synteny in the Epsilonproteobacteria. All protein-coding annotated features of 42 species of Epsilonproteobacteria (Additional file 14: Table S7) were compared by pairwise BLASTP against *C. jejuni* NCTC 11168 (pages 1–2) and *H. pylori* 26695 (pages 3–4). The highest scoring ortholog in the pairwise comparison was used if the E-score was > 1 E-06, and used in a scatter plot [77]. The Gamma-proteobacteria *E. coli* and *Thiomicrospora crunogena* are included for comparison. An overview of the total number of genes orthologous between these species is given in Additional file 16: Table S8. Click here for file

Additional file 16: Table S8 Comparative genomics of selected Epsilonproteobacteria. Click here for file

Additional file 17: Table S9 Prediction of putative leaderless transcripts in the Epsilonproteobacteria. Click here for file

Additional file 18: Table S10 Conservation of internal promoters in the Epsilonproteobacteria. Click here for file

Additional file 19: Figure S9 Conservation of internal promoters is partially dependent on conservation of gene order, whereas antisense RNAs show little conservation within the Epsilonproteobacteria. A) Schematic overview of conservation of internal promoters in the Epsilonproteobacteria, based on promoter predictions shown in Additional file 18: Table S10. For most of the predicted internal promoter, there is a link with the presence of the downstream orthologous gene(s), suggesting evolutionary pressure on the transcriptional circuitry. B) Antisense promoters differing in location between *C. jejuni* and *H. pylori* do not show conservation (as shown for the *gyrB* gene, see Figure 3C), whereas antisense promoters conserved between *C. jejuni* and *H. pylori* are predicted to be present in the majority of Epsilonproteobacteria (as shown for the *clpB* gene, see Figure 3C). Full information of the promoter predictions can be found in Additional file 20: Table S11. Click here for file

Additional file 20: Table S11 Conservation of antisense promoters in the Epsilonproteobacteria. Click here for file

Additional file 21: Table S12 Prediction of putative leader peptides in the Epsilonproteobacteria. Click here for file

Additional file 22: Table S13 Oligonucleotides used in this study. Click here for file

Additional file 23 **Artemis entry file for use with the *C. jejuni* NCTC 11168 genome sequence (accession number NC_002163).** This file contains colour-coded entries of each TSS and promoter sequence from -50 to +1, with annotation as given in Additional file 3: Table S1. Click here for file
