# Supplementary figures and images for: Parallel evolution of genome structure and transcriptional landscape in the Epsilonproteobacteria
Source: BMC Genomics. 2013 Sep 12;14:616. doi: 10.1186/1471-2164-14-616 (PMC3847290; doi:10.1186/1471-2164-14-616)

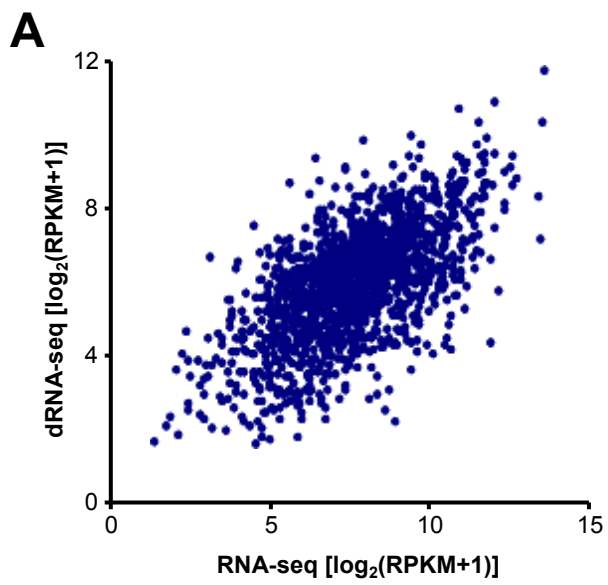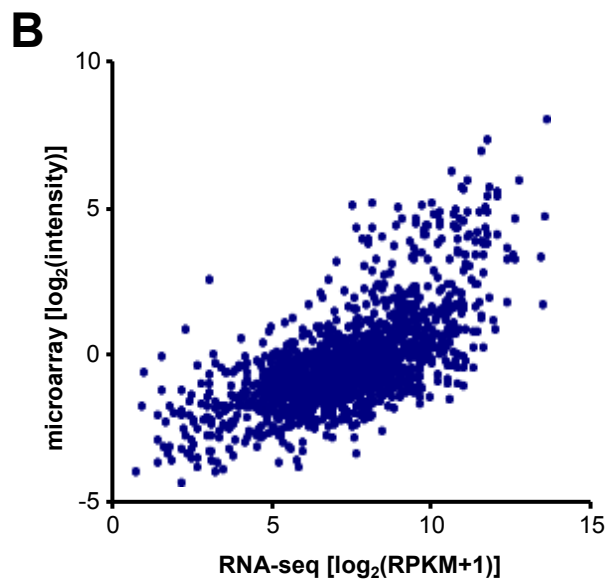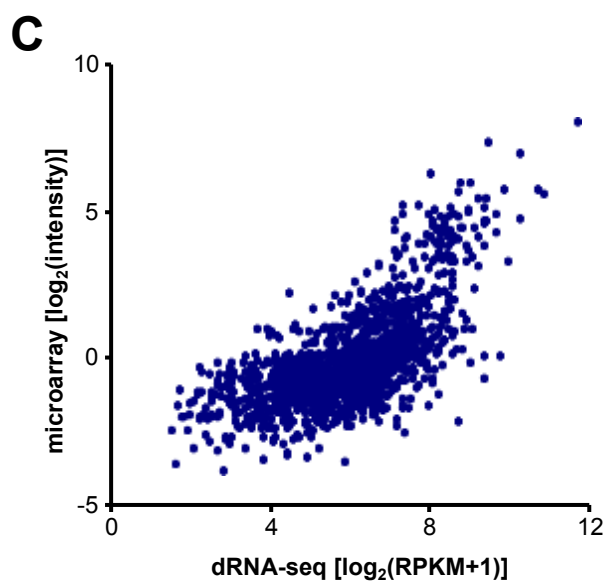

Supplement: Additional file 1: Figure S1 — Comparison of expression levels of C. jejuni genes on RNA-seq and microarray, using the Illumina-based quantitative RNA-seq data from Chaudhuri et al. [21], with and microarray and differential RNA-seq data (this study). A) Comparison of RNA-seq and the -TEX reads from differential RNA-seq analysis, based on log2(RPKM + 1) values [21] for 1509 genes. B) Comparison of RNA-seq [21] and normalised microarray expression levels for 1542 genes. C) Comparison of differential RNA-seq and normalised microarray expression levels for 41423 genes. [file 1471-2164-14-616-S1.pdf]

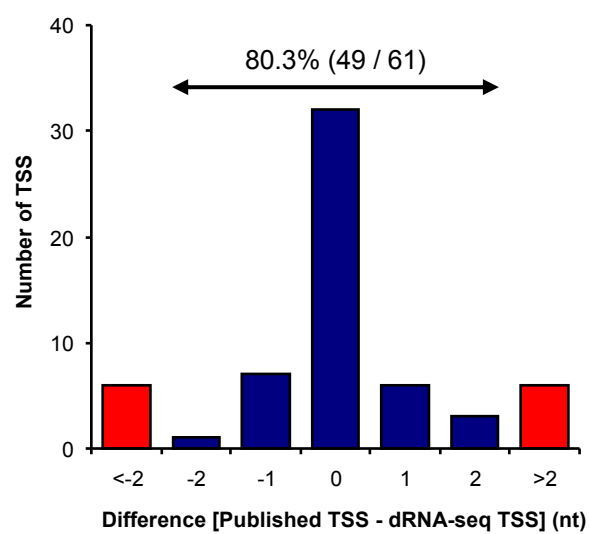

Supplement: Additional file 6: Figure S3 — Comparison of C. jejuni TSS identified by dRNA-seq with those previously published (Additional file 5: Table S3). The histogram indicates the number of distances between 61 TSS identified in C. jejuni by primer extension and 5′ RACE with those determined by dRNA-seq. [file 1471-2164-14-616-S6.pdf]

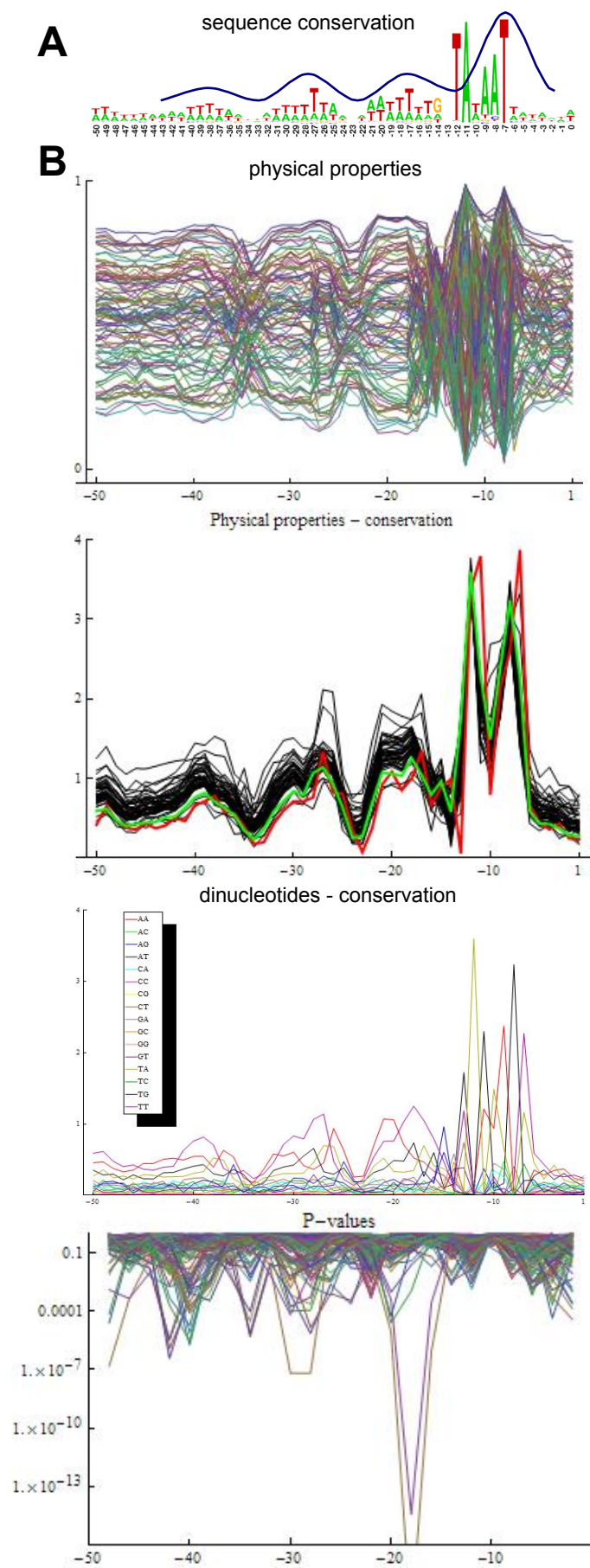

Supplement: Additional file 7: Figure S4 — Sequence conservation in C. jejuni σ70 promoters is matched by conservation in physico-chemical properties. A) WebLogo representation of the −50 to +1 sequences of σ70 promoters in C. jejuni (Additional file 3: Table S1). B) Profiles for 99 physical DNA properties taken from DiProDB [36] (upper panel), conservation of dinucleotides (panel 2) and conservation of 99 physical properties [panel 3; for comparison: red curve nucleotide conservation (corresponding to height of weblogo), green dinucleotide conservation]. The two curves peaking at −26, −27 are slide and entropy. The lower panel shows the significance of correlation of physical properties of neighboured dinucleotides (uncorrected p-values). The two curves peaking at −18 are inclination and direction of the deflection angle. [file 1471-2164-14-616-S7.pdf]

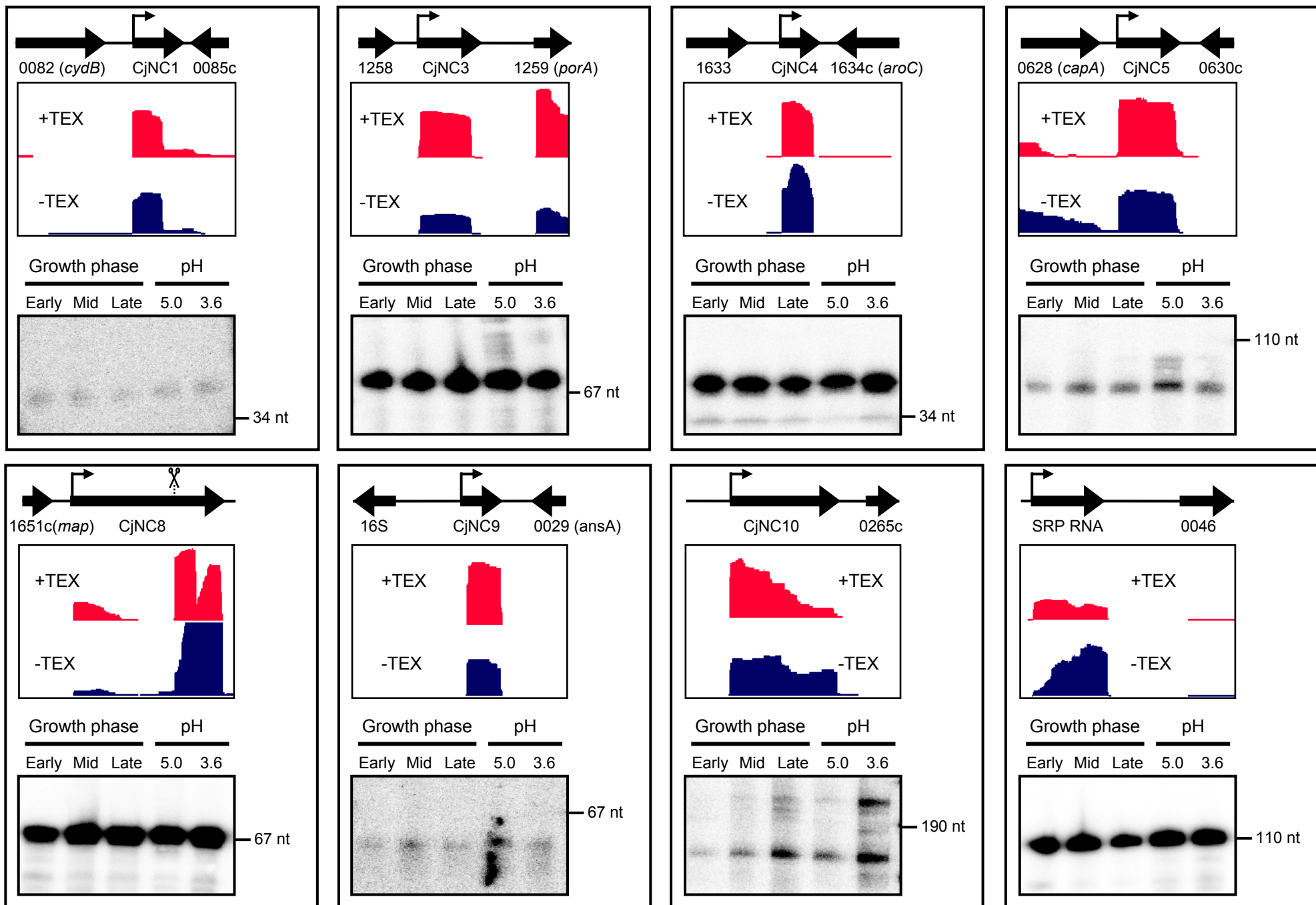

Porcelli et al , Figure S5

Supplement: Additional file 10: Figure S5 — Identification of non-coding RNAs (ncRNAs) in the C. jejuni transcriptome and independent confirmation of their transcription using Northern hybridisation. For 7 ncRNAs, the dRNA-seq histograms are shown, with the red histograms representing the + TEX cDNA library enriched for primary transcripts, and the blue histograms representing the non-enriched -TEX cDNA library. Genes/ncRNAs are shown above the histograms with the arrows representing their transcriptional direction, while small arrows indicate the position of transcription start sites and orientation of promoters. Below the histograms, Northern hybridisations are shown with independent RNA-samples, isolated in early, mid and late log growth phases, and after 30 minutes exposure to pH 5.0 and pH 3.6. Relevant marker sizes are indicated on the right hand side. The scissor symbol above the CjNC8 ncRNA indicates a putative post-transcriptional modification site, resulting in a mature RNA of 70 nt. The SRP RNA is included as control. Full information on the ncRNAs can be found in Additional file 9: Table S5. [file 1471-2164-14-616-S10.pdf]

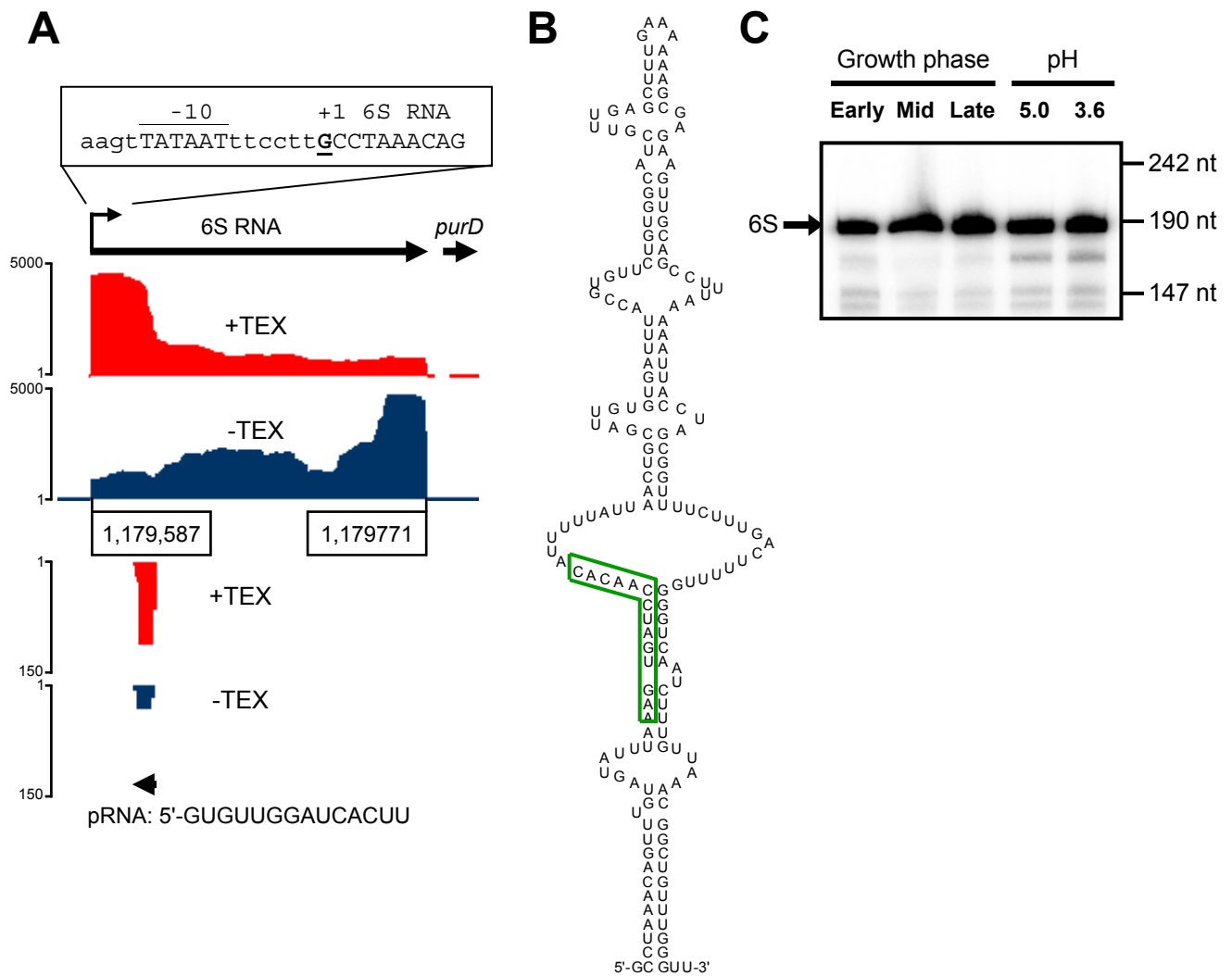

Supplement: Additional file 11: Figure S6 — Identification and characterisation of the C. jejuni 6S RNA. (A) The 6S RNA is encoded directly upstream of the purD (cj1250) gene, and is transcribed on the leading strand from a σ70 promoter upstream of the TSS (shown above the histograms). The product RNA (pRNA) transcribed from the complimentary strand is shown below. (B) Predicted folding of the C. jejuni 6S RNA. The sequence used to transcribe the complementary pRNA is marked by a green box. (C) Transcription of the 185 nt 6S RNA is constitutive during exponential growth and during acid shock, as demonstrated using Northern hybridisation. [file 1471-2164-14-616-S11.pdf]

**A**

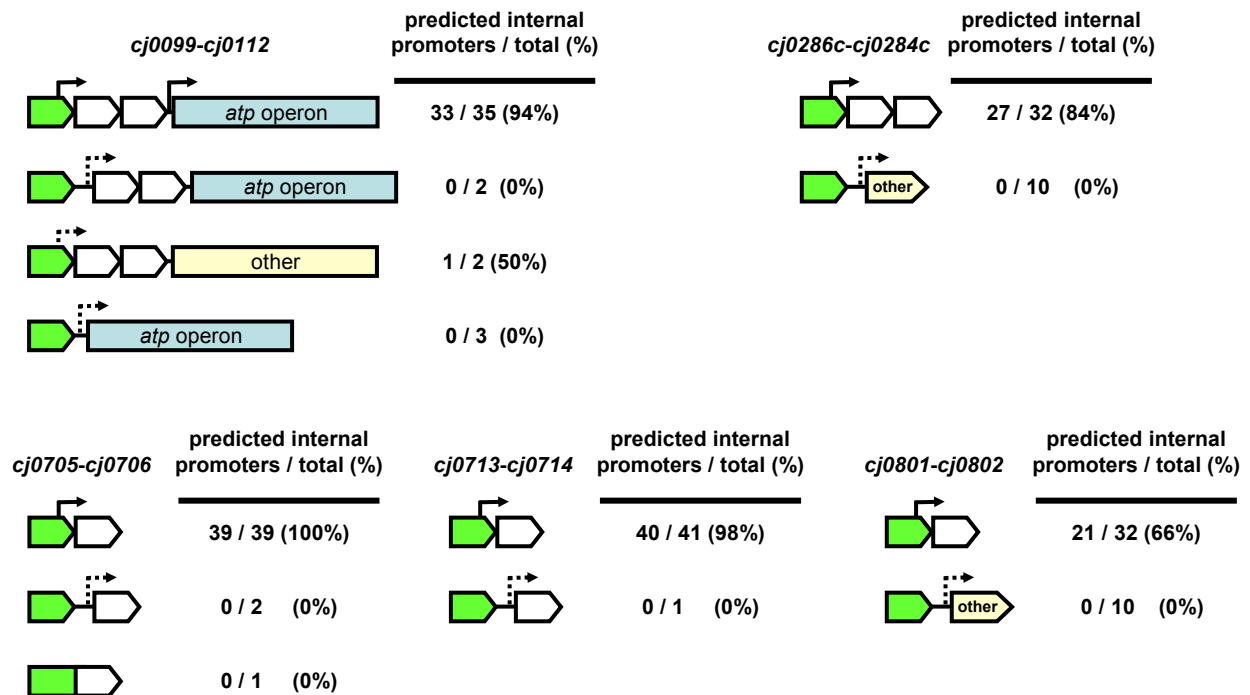

**B**

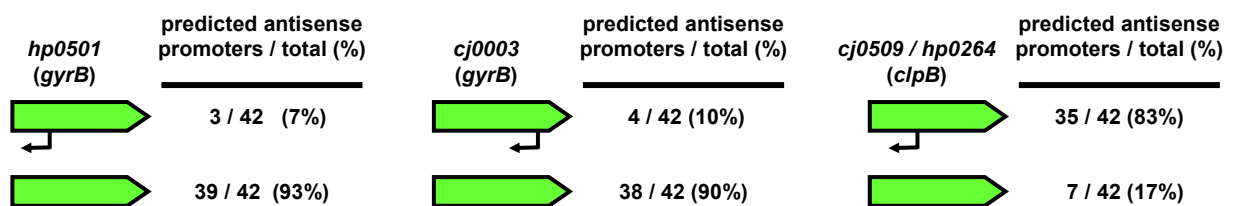

Supplement: Additional file 19: Figure S9 — Conservation of internal promoters is partially dependent on conservation of gene order, whereas antisense RNAs show little conservation within the Epsilonproteobacteria. A) Schematic overview of conservation of internal promoters in the Epsilonproteobacteria, based on promoter predictions shown in Additional file 18: Table S10. For most of the predicted internal promoter, there is a link with the presence of the downstream orthologous gene(s), suggesting evolutionary pressure on the transcriptional circuitry. B) Antisense promoters differing in location between C. jejuni and H. pylori do not show conservation (as shown for the gyrB gene, see Figure 3C), whereas antisense promoters conserved between C. jejuni and H. pylori are predicted to be present in the majority of Epsilonproteobacteria (as shown for the clpB gene, see Figure 3C). Full information of the promoter predictions can be found in Additional file 20: Table S11. [file 1471-2164-14-616-S19.pdf]
