# Supplementary material for: Parallel evolution of genome structure and transcriptional landscape in the Epsilonproteobacteria
Source: BMC Genomics. 2013 Sep 12;14:616. doi: 10.1186/1471-2164-14-616 (PMC3847290; doi:10.1186/1471-2164-14-616)
Supplement: Additional file 2: Figure S2 — Identification of transcription start sites in C. jejuni. A) Schematic representation of the different types of transcription start sites, with primary and secondary TSS being located at ≤ 500 nt from the translational startcodon of the respective gene. TSS can have multiple associations, as shown for the primary and internal TSS within the first gene. B) Venn diagram representing the overlap between the different classes of TSS identified for C. jejuni. C) The cj1316c is transcribed from both a primary and secondary TSS (left) whereas the ftsZ (cj0696) gene is transcribed from an internal promoter located in the coding sequence of the upstream ftsA (cj0695) gene (right), allowing intraoperonic differentiation of transcript levels. Translational start codons and putative RBS are underlined, TSS are shown underlined in bold typeface, and extended and normal −10 sequences for σ70 and σ28 are indicated in bold typeface. [file 1471-2164-14-616-S2.pdf]

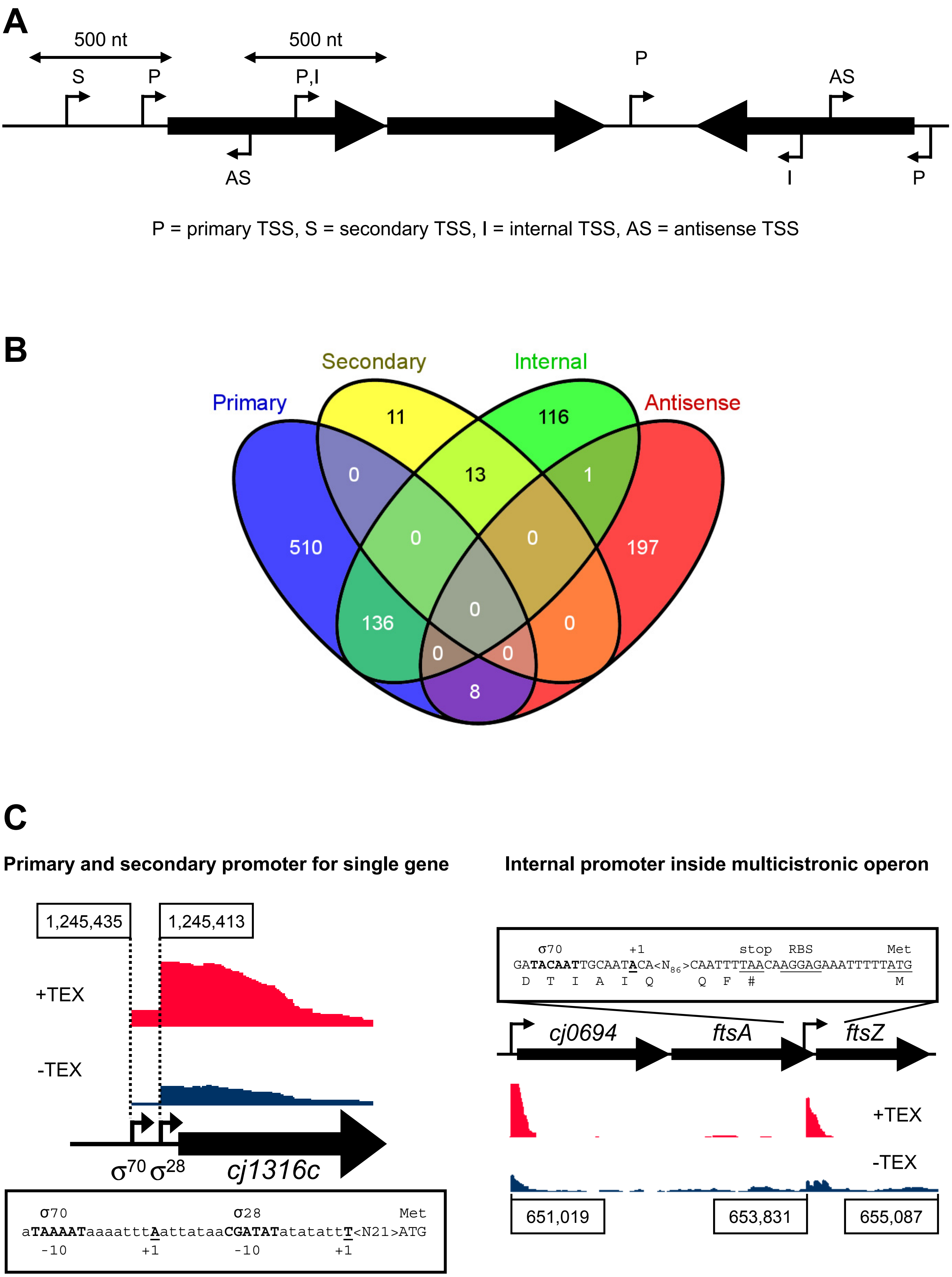

Porcelli et al , Figure S2
