# Supplementary material for: Parallel evolution of genome structure and transcriptional landscape in the Epsilonproteobacteria
Source: BMC Genomics. 2013 Sep 12;14:616. doi: 10.1186/1471-2164-14-616 (PMC3847290; doi:10.1186/1471-2164-14-616)
Supplement: Additional file 4: Table S2 — C. jejuni genes with primary and secondary promoters. [file 1471-2164-14-616-S4.pdf]

Table S2. *C. jejuni* genes with primary and secondary promoters.

Gene	Promoter sequences from upstream -26 to ATG. Sigma factor recognition sequences are indicated [a]
Cj0054c	acctattaaaactta**TATAAT**aaaga**<u>A</u>**cttat<N10>ta**gcTAGAAT**ggaat**<u>T</u>**tattttaactaAAGGttgaaaaATG
	σ70 σ70
Cj0102	tttagaaaatatt**gaTAAAAT**taaaa**<u>A</u>**att<N40>ttttat**ggTAAAAT**agtcct**<u>T</u>**ca<N10>ccaAGGAgaattATG
	σ70 σ70
Cj0205	**GGCA**cgcttttt**TGCTT**cagggcttat**<u>A</u>**gtg<N212>ttct**gaTATAAT**ttttc**<u>T</u>**tttgc<N10>caAGGAttaaaATG
	σ54 σ70
Cj0342c	tatccaagttcttt**TAATAT**tattat**<u>G</u>**c**TAAAAT**cttaaa**<u>T</u>**ttatttaatgtttAGGAaagactATG
	σ70 σ70
Cj0476	aagaacaattat**ggGATAAT**gttagc**<u>A</u>**ctttt<N150>gttt**TAAAAT**aaacat**<u>T</u>**ga<N95>aaAGGAgaaaatGTG
	σ70 σ70
Cj0547	**GGCA**cagtttt**TGCTT**attattttt**<u>T</u>**tatta<N45>ttgt**CGATAT**aagctttt**<u>A</u>**ac<N20>aAGGAttttaaaATG
	σ54 σ28
Cj0897c	tattgttggttt**gaTACAAT**tatatc**<u>T</u>**ttaa<N35>aa**TAGAAT**tagaac**<u>T</u>**tga<N10>ttAAGGtaagaaaaTTG
	σ70 σ70
Cj1001	ataaacataataac**CGATTT**atatag**<u>A</u>**aagtt<N10>ta**gaTAAAAT**ccaaa**<u>G</u>**ctt<N10>caAGGAtaatcaATG
	σ28 σ70
Cj1103	aaagcttaaatgt**gtTAAAAT**acatt**<u>A</u>**accta<N135>aatacag**TAAAAT**atcttct**<u>A</u>**aatAAGGttgaaaaATG
	σ70 σ70
Cj1156	ttaaaaaaatattttt**TATAAT**tata**<u>A</u>**aca<N25>at**TAAAAT**caca**<u>T</u>**tttac<N20>ttacGAGGacttATG
	σ70 σ70
Cj1316c	atatataaaatca**TAAAAT**aaaattt**<u>A</u>**attataa**CGATAT**atatattt**<u>A</u>**agactaattaaAGGAaatttATG
	σ70 σ28
Cj1361c	aataagatttta**gcTAAAAT**tt**gcTA<u>G</u>AAT**ttacatt**<u>A</u>**aactttaatgattATGGtttaaaaATG
	σ70 σ70

a) Promoter sequences indicated are: σ^{28}, CGATwT at -10 position; σ^{54}, GGcA-N7-TGCTT at -24/-12 positions; σ^{70}, gnTAnaAT at -10 position. All relevant sequences are shown in capital letters; bold typeface indicates promoter sequences, bold and underlined typeface indicate TSS, capital letters alone indicate ribosome binding sites and startcodons. <Nxx> indicates length of sequences not shown.
