# Supplementary material for: Parallel evolution of genome structure and transcriptional landscape in the Epsilonproteobacteria
Source: BMC Genomics. 2013 Sep 12;14:616. doi: 10.1186/1471-2164-14-616 (PMC3847290; doi:10.1186/1471-2164-14-616)
Supplement: Additional file 5: Table S3 — Comparison of C. jejuni TSS identified by dRNA-seq with TSS identified by 5′ RACE and primer extension. [file 1471-2164-14-616-S5.pdf]

Table S3. Comparison of *C. jejuni* TSS identified by dRNA-seq with TSS identified by 5' RACE and primer extension

Cj gene	gene name and location TSS	*C. jejuni* strain	Strand	Published TSS	dRNA-seq TSS	Difference [a]	Method [b]	Reference
0008	hypothetical, antisense	NCTC 11168	-	13687	13687	0	RACE	This study
0062c	hypothetical	81-176	-	76518	76614	96	PE	[1]
0069	hypothetical	129108	+	81996	81995	-1	PE	[2]
0076c	*lctP*	NCTC 11168	-	88746	88746	0	RACE	This study
0079c	*cdtA*	81116	-	91151	91099	-52	PE	[3]
0169	*sodB*	129108	+	166345	166345	0	PE	[2]
0193c	*tig*	129108	-	189481	189481	0	PE	[2]
0200c	hypothetical	81116	-	196493	196494	1	PE	[4]
0322	*perR*	NCTC 11168	+	293905	293907	2	PE	[5]
0333c	*fdxA*	81116	-	302204	302204	0	PE	[6]
0334	*ahpC*	81116	+	302352	302353	1	PE	[7]
0335	*flhB*	81116	+	303008	303066	58	PE	[8]
0369c	hypothetical	NCTC 11168	-	338941	338946	5	PE	[9]
0437_up	*mfrX*	NCTC 11168	+	405091	405091	0	RACE	[10]
0465c	*ctb*	NCTC 11168	-	430983	430983	0	PE	[11]
0509c	*clpB*	clinical isolate	-	477261	477260	-1	PE	[12]
0531	*icd*	129108	+	494044	494044	0	PE	[2]
0606	hypothetical	81116	+	564447	564447	0	PE	[4]
0613	*pstS*	81116	+	573200	573199	-1	PE	[13]
0671	*dcuB*, antisense	NCTC 11168	-	626800	626800	0	RACE	This study
0693c	hypothetical	129108	-	650905	650903	-2	PE	[2]
0696	*ftsZ*, internal	NCTC 11168	-	653831	653831	0	RACE	This study
0757	*hcrA*	clinical isolate	+	707806	707802	-4	PE	[14]
0762c	*aspB*	129108	-	713986	713988	2	PE	[2]
0830	hypothetical	NCTC 11168	+	776863	776863	0	RACE	[11]
0883c	hypothetical	129108	-	820178	820179	1	PE	[2]
0884	*rpsO*	129108	+	820287	820287	0	PE	[2]
0886c	*ftsK*	129108	-	823523	823609	86	PE	[2]
0913c	*hupB*	129108	-	849758	849758	0	PE	[2]
0924c	*cheB'*	129108	-	860168	860162	-6	PE	[2]
0989	hypothetical	129108	+	921165	921114	-51	PE	[2]
1034c	*fedC*	81-176	-	969401	969400	-1	PE	[1]

1061c	*ileS*	129108	-	999554	999554	0	PE	[2]
1064	*rdxA*	81-176	+	1001190	1001190	0	PE	[15]
1096c	*metK*	129108	-	1030835	1030836	1	PE	[2]
1103	*csrA*, internal	NCTC 11168	-	1037934	1037934	0	RACE	This study
1103	*csrA*, internal	NCTC 11168	-	1038095	1038095	0	RACE	This study
1132c	hypothetical	HB93-13	-	1066758	1066758	0	RACE	[16]
1133	*waaC*	HB93-13	+	1066773	1066773	0	RACE	[16]
1146c	*waaV*	HB93-13	-	1080876	1080878	2	RACE	[16]
1148	*waaF*	HB93-13	+	1080877	1080878	1	RACE	[16]
1156	*rho*	129108	+	1088330	1088294	-36	PE	[2]
1220	*groES*	clinical isolate	+	1149145	1149145	0	PE	[17]
1223c	*dccR*	81116	-	1153040	1153039	-1	PE	[4]
1288c	*gltX2*	129108	-	1221396	1221408	12	PE	[2]
1293	*pseB*	81-176	+	1224822	1224822	0	PE	[18]
1338c	*flaB*	81116	-	1269112	1269112	0	PE	[19]
1339c	*flaA*	81116	-	1270993	1270994	1	PE	[19]
1355	*ceuE*, antisense	NCTC 11168	-	1286962	1286962	0	RACE	This study
1356c	hypothetical	81116	-	1288473	1288472	-1	PE	[4]
1450	*ciaI*	81-176	+	1388231	1388235	4	PE	[1]
1500	*fdhT*	NCTC 11168	+	1432561	1432561	0	RACE	[20]
1503c	*putP*	NCTC 11168	-	1439066	1439066	0	RACE	[20]
1505c	hypothetical	NCTC 11168	-	1440795	1440795	0	RACE	[20]
1506c	*cca*	NCTC 11168	-	1443012	1443012	0	RACE	[20]
1508c	*fdhD*	NCTC 11168	-	1444629	1444629	0	RACE	[20]
1514c	*fdhM*	NCTC 11168	-	1450118	1450118	0	RACE	[20]
1558	*arsP*	CB5-28	+	1490186	1490183	-3	PE	[21]
1586	*cgb*	NCTC 11168	+	1517544	1517544	0	RACE	[11]
1615	*chuB*, antisense	NCTC 11168	-	1543051	1543051	0	RACE	This study
1626c	hypothetical	81116	-	1555084	1555083	-1	PE	[4]

a) Difference is expressed as [Published TSS - dRNA-seq TSS].

b) PE = primer extension analysis, RACE = 5' RACE (Rapid extension of cDNA ends).

REFERENCES

1. Barrero-Tobon AM, Hendrixson DR: **Identification and analysis of flagellar coexpressed determinants (Feds) of Campylobacter jejuni involved in colonization**. *Mol Microbiol* 2012, **84**(2):352-369.
2. Wosten MM, Boeve M, Koot MG, van Nuenen AC, van der Zeijst BA: **Identification of Campylobacter jejuni promoter sequences**. *J Bacteriol* 1998, **180**(3):594-599.
3. Jeon B, Itoh K, Ryu S: **Promoter analysis of cytolethal distending toxin genes (cdtA , B, and C) and effect of a luxS mutation on CDT production in Campylobacter jejuni**. *Microbiol Immunol* 2005, **49**(7):599-603.
4. Wosten MM, van Dijk L, Parker CT, Guilhabert MR, van der Meer-Janssen YP, Wagenaar JA, van Putten JP: **Growth phase-dependent activation of the DccRS regulon of *Campylobacter jejuni***. *J Bacteriol* 2010, **192**(11):2729-2736.
5. Kim M, Hwang S, Ryu S, Jeon B: **Regulation of perR expression by iron and PerR in Campylobacter jejuni**. *J Bacteriol* 2011:Epub ahead of print, 13 September 2011.
6. van Vliet AHM, Baillon MA, Penn CW, Ketley JM: **The iron-induced ferredoxin FdxA of Campylobacter jejuni is involved in aerotolerance**. *FEMS Microbiol Lett* 2001, **196**(2):189-193.
7. Baillon ML, van Vliet AHM, Ketley JM, Constantinidou C, Penn CW: **An iron-regulated alkyl hydroperoxide reductase (AhpC) confers aerotolerance and oxidative stress resistance to the microaerophilic pathogen Campylobacter jejuni**. *J Bacteriol* 1999, **181**(16):4798-4804.
8. Matz C, van Vliet AHM, Ketley JM, Penn CW: **Mutational and transcriptional analysis of the Campylobacter jejuni flagellar biosynthesis gene flhB**. *Microbiology* 2002, **148**(Pt 6):1679-1685.
9. Guo B, Wang Y, Shi F, Barton YW, Plummer P, Reynolds DL, Nettleton D, Grinnage-Pulley T, Lin J, Zhang Q: **CmeR functions as a pleiotropic regulator and is required for optimal colonization of Campylobacter jejuni in vivo**. *J Bacteriol* 2008, **190**(6):1879-1890.
10. Guccione E, Hitchcock A, Hall SJ, Mulholland F, Shearer N, van Vliet AHM, Kelly DJ: **Reduction of fumarate, mesaconate and crotonate by Mfr, a novel oxygen-regulated periplasmic reductase in Campylobacter jejuni**. *Environ Microbiol* 2010, **12**(3):576-591.
11. Elvers KT, Turner SM, Wainwright LM, Marsden G, Hinds J, Cole JA, Poole RK, Penn CW, Park SF: **NssR, a member of the Crp-Fnr superfamily from Campylobacter jejuni, regulates a nitrosative stress-responsive regulon that includes both a single-domain and a truncated haemoglobin**. *Mol Microbiol* 2005, **57**(3):735-750.
12. Thies FL, Karch H, Hartung HP, Giegerich G: **The ClpB protein from Campylobacter jejuni: molecular characterization of the encoding gene and antigenicity of the recombinant protein**. *Gene* 1999, **230**(1):61-67.
13. Wosten MM, Parker CT, van Mourik A, Guilhabert MR, van Dijk L, van Putten JP: **The Campylobacter jejuni PhosS/PhosR operon represents a non-classical phosphate-sensitive two-component system**. *Mol Microbiol* 2006, **62**(1):278-291.
14. Thies FL, Karch H, Hartung HP, Giegerich G: **Cloning and expression of the dnaK gene of Campylobacter jejuni and antigenicity of heat shock protein 70**. *Infect Immun* 1999, **67**(3):1194-1200.
15. Ribardo DA, Bingham-Ramos LK, Hendrixson DR: **Functional Analysis of the RdxA and RdxB Nitroreductases of Campylobacter jejuni Reveals Mutations in rdxA Confer Metronidazole Resistance**. *J Bacteriol* 2010, **192**(7):1890-1901.

16. Phongsisay V, Fry BN: **Bidirectional transcription of lipooligosaccharide synthesis genes from Campylobacter jejuni**. *Int J Med Microbiol* 2007, **297**(6):431-441.
17. Thies FL, Weishaupt A, Karch H, Hartung HP, Giegerich G: **Cloning, sequencing and molecular analysis of the Campylobacter jejuni groESL bicistronic operon**. *Microbiology* 1999, **145 (Pt 1)**:89-98.
18. Goon S, Kelly JF, Logan SM, Ewing CP, Guerry P: **Pseudaminic acid, the major modification on Campylobacter flagellin, is synthesized via the Cj1293 gene**. *Mol Microbiol* 2003, **50**(2):659-671.
19. Nuijten PJ, van Asten FJ, Gaastra W, van der Zeijst BA: **Structural and functional analysis of two Campylobacter jejuni flagellin genes**. *J Biol Chem* 1990, **265**(29):17798-17804.
20. Shaw FL, Mulholland F, Le Gall G, Porcelli I, Hart DJ, Pearson BM, van Vliet AHM: **Selenium-dependent biogenesis of formate dehydrogenase in Campylobacter jejuni is controlled by the fdhTU accessory genes**. *J Bacteriol* 2012, **194**(15):3814-3823.
21. Wang L, Jeon B, Sahin O, Zhang Q: **Identification of an arsenic resistance and arsenic-sensing system in Campylobacter jejuni**. *Appl Environ Microbiol* 2009, **75**(15):5064-5073.
