# Supplementary material for: Parallel evolution of genome structure and transcriptional landscape in the Epsilonproteobacteria
Source: BMC Genomics. 2013 Sep 12;14:616. doi: 10.1186/1471-2164-14-616 (PMC3847290; doi:10.1186/1471-2164-14-616)
Supplement: Additional file 9: Table S5 — C. jejuni non-coding RNAs in intergenic regions. [file 1471-2164-14-616-S9.pdf]

**Table S5. *C. jejuni* non-coding RNAs in intergenic regions**

| Name | Strand | TSS | 3' end | length | Left | orientation | Right | Comments [a] |
|---|---|---|---|---|---|---|---|---|
| CjNC9 [b] | - | 38730 | 38679 | 52 | Cj0029 | ▶ ◀ ▶ | 16S rRNA | |
| SRP RNA | + | 66641 | 66748 | 108 | Cj0045c | ◀ ▶ ▶ | Cj0046 | SRP |
| CjNC11 [b] | - | 83265 | 83111 | 155 | Cj0069 | ▶ ◀ ▶ | Cj0072c | |
| CjNC1 | + | 94249 | 94293 | 45 | Cj0082 | ▶ ▶ ◀ | Cj0085c | CJnc10 |
| CjNC10 | - | 245380 | 245225 | 156 | Cj0265c | ◀ ◀ ◀ | Cj0266c | CJnc20 |
| TPP | + | 418415 | 418550 | 136 | Cj0452 | ▶ ▶ ▶ | Cj0453 | TPP riboswitch |
| CjNC20 [b] | + | 587562 | 587617 | 56 | Cj0627 | ▶ ▶ ▶ | Cj0628 | |
| CjNC5 | + | 591313 | 591426 | 114 | Cj0628 | ▶ ▶ ◀ | Cj0630c | CJnc30 |
| CjNC21 | - | 591456 | 591356 | 101 | Cj0628 | ▶ ◀ ◀ | Cj0630c | CJnc40 |
| CjNC12 [b] | - | 613202 | 613072 | 131 | Cj0652 | ▶ ◀ ◀ | Cj0653c | |
| CjNC22 [b] | - | 707581 | 707553 | 29 | Cj0755 | ▶ ◀ ▶ | Cj0757 | |
| CjNC19 [b] | + | 878078 | 878135 | 58 | Cj0939c | ◀ ▶ ◀ | tRNA-Asp | antisense to tRNA Asp |
| CjNC14 [b] | + | 1029434 | 1029534 | 101 | Cj1095 | ▶ ▶ ◀ | Cj1096c | |
| CjNC15 | + | 1127993 | 1128130 | 138 | Cj1198 | ▶ ▶ ▶ | Cj1199 | CJnc110 |
| CjNC18 [b] | - | 1128196 | 1128061 | 136 | Cj1198 | ▶ ◀ ▶ | Cj1199 | antisense to CjNC15 |
| 6S RNA | + | 1179587 | 1179771 | 185 | Cj1249 | ▶ ▶ ▶ | Cj1250 | |
| pRNA | - | 1179623 | 1179610 | 14 | Cj1249 | ▶ ◀ ▶ | Cj1250 | antisense to 6S RNA |
| CjNC3 | + | 1188925 | 1188997 | 73 | Cj1258 | ▶ ▶ ▶ | Cj1259 | CJnc140 |
| 10Sa RNA | - | 1293658 | 1293301 | 358 | Cj1359 | ▶ ◀ ◀ | Cj1361c | tmRNA, Cj1360c |
| CRISPR_1 | + | 1455167 | 1455204 | 38 | Cj1519 | ▶ ▶ ◀ | Cj1521c | crRNA1 |
| CRISPR_2 | + | 1455232 | 1455269 | 38 | Cj1519 | ▶ ▶ ◀ | Cj1521c | crRNA2 |
| CRISPR_3 | + | 1455299 | 1455336 | 38 | Cj1519 | ▶ ▶ ◀ | Cj1521c | crRNA3 |
| CRISPR_4 | + | 1455365 | 1455403 | 39 | Cj1519 | ▶ ▶ ◀ | Cj1521c | crRNA4 |
| CRISPR_5 | + | 1455498 | 1455570 | 73 | Cj1519 | ▶ ▶ ◀ | Cj1521c | tracrRNA |
| CjNC4 | + | 1559676 | 1559722 | 47 | Cj1633 | ▶ ▶ ◀ | Cj1634c | CJnc170 |
| CjNC16 | + | 1575015 | 1575112 | 98 | Cj1650 | ▶ ▶ ◀ | Cj1651c | CJnc180, antisense CjNC8 |
| CjNC8 | - | 1575257 | 1575042 | 215 (70) | Cj1650 | ▶ ◀ ◀ | Cj1651c | CJnc190, processed RNA [b] |
| CjNC6 | + | 1600382 | 1600468 | 87 | Cj1677 | ▶ ▶ ▶ | Cj1679 | CJnc210 |
| CjNC23 | - | 1638039 | 1637942 | 98 | Cj1727c | ◀ ◀ ◀ | Cj1729c | CJnc230 |

a) Column includes sRNA nomenclature proposed by [1].

b) Absent from sRNA list in [1].

c) CjNC8 seems to be a processed ncRNA, which has its TSS at position 1575042, but is processed to a mature RNA starting at 1575111 (length 70 nt).

Reference:

1. Dugar G, Herbig A, Forstner KU, Heidrich N, Reinhardt R, Nieselt K, Sharma CM: **High-Resolution Transcriptome Maps Reveal Strain-Specific Regulatory Features of Multiple Campylobacter jejuni Isolates**. *PLoS Genet* 2013, **9**(5):e1003495.
