# Supplementary material for: Parallel evolution of genome structure and transcriptional landscape in the Epsilonproteobacteria
Source: BMC Genomics. 2013 Sep 12;14:616. doi: 10.1186/1471-2164-14-616 (PMC3847290; doi:10.1186/1471-2164-14-616)
Supplement: Additional file 12: Figure S7 — Identification of C. jejuni putative leader peptides allowing coupling of amino acid availability to downstream expression of the amino acid biosynthetic pathways for tryptophan, methionine and leucine. The sequence of the leader peptide is indicated, with the corresponding regulatory amino acid in red typeface. When similar leader peptides are predicted to be present in other Epsilonproteobacteria (based on location and presence of a ribosome binding site), their sequence is included. The dRNA-seq histograms for C. jejuni are shown, with the red histograms representing the + TEX cDNA library enriched for primary transcripts, and the blue histograms representing the non-enriched -TEX cDNA library. [file 1471-2164-14-616-S12.pdf]

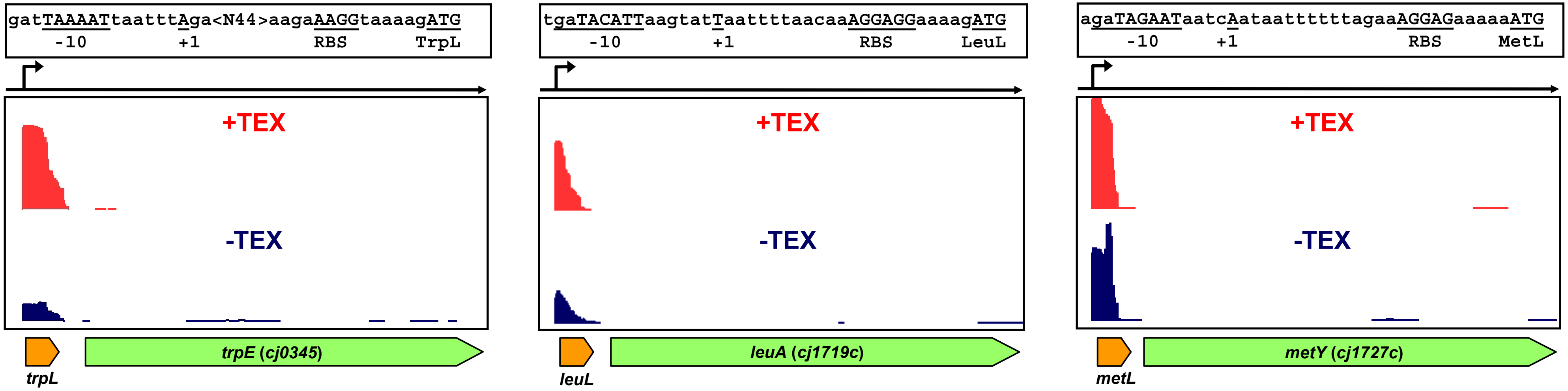

Cjejuni	: MSENGFFSNSLILKLTHTLRSWRF	: MKQIFEISNSVQQKSKDNTFNLLLQLSL	: MSYISFNLMLSSFCMRAWHA
Cdoylei	: MSENDFFSNSLILKLTHTLRSWCF	: MKQIFKISNSVQQKSKDNTFNLLLQLSL	: MSYTSFNFMLSSFCMRAWHA
Ccoli	: MSENDFFSNSLILKLTHTLRSWCF	: MTQV<X11>QKNKENHFNLMLQLSLILN	: MSYISFNLMLSSFCMRAWHA
Clari	: MERKKFSKLILEYSFNKASFSWR	: n/a	: n/a
Cupsaliens	: --	: n/a	: n/a
Cfetus	: --	: MTTPFSQKKYIFIYKLSLLNLL	: --
Cconcicus	: --	: MSENGIIKSRKFLPKIEIKNSLLLLR	: --
Ccurvus	: --	: MSDAGIIKSRKFLPKIEIKNSLLLLW	: --
Crectus	: n/a	: MNGNVKFNAFFSLLLLLR	: --
Cshowae	: --	: MNGNVKFNAFFSLLLLLR	: --
Cgracilis	: --	: --	: --
Chominis	: n/a	: --	: --
Sulfdeleyi	: --	: MTCLSRFKISDQKKNNTLLLLLLLNF	: --
Arcbutzler	: --	: MKIFDFYKSHFIKSVNSLLLLSFSL	: --
Wolsuccino	: --	: MTSV<X11>NTFNIKIELSLLPLLLLSL	: --
Hmustelae	: --	: MKNKFFLSFSVFYLSPLLQF	: n/a
Hhepaticus	: --	: LTIQSDTLRFSYNLKAFVFREFLLLM	: --
Hcinaedi	: --	: --	: --
Hpylori	: --	: n/a	: --
Hfelis	: --	: n/a	: --
Hbizzozero	: --	: n/a	: --
Sulkujiens	: --	: MKSHTMPISQPITDNRIVSFSLLLLPLL	: --
Sudenitrif	: --	: MIPFNMMSLLSFILVTKPLFL	: --
Sulfurovum	: --	: MKTIRIHTTIFTKDCSLLLLLAF	: --
Nitratirup	: --	: VSETGVIKAQKYLPKIEIKNHLLHLL	: --
Nautprofun	: --	: --	: --

Porcelli et al, Figure S7
