# Supplementary material for: Parallel evolution of genome structure and transcriptional landscape in the Epsilonproteobacteria
Source: BMC Genomics. 2013 Sep 12;14:616. doi: 10.1186/1471-2164-14-616 (PMC3847290; doi:10.1186/1471-2164-14-616)
Supplement: Additional file 13: Table S6 — Leaderless mRNAs of C. jejuni NCTC 11168. [file 1471-2164-14-616-S13.pdf]

Table S6: Leaderless mRNAs of *C. jejuni* NCTC 11168

gene [a]	strand	TSS	Start	UTR length	RBS	Leaderless	Sequence -30 to startcodon [b]
cj0153c	-	157298	157298	0	no	yes	ttttaagtcaagatta**ggTAAAAT**tttaat**A**TG
cj0164c	-	164091	164091	0	no	yes	tcatagcaaaaaatattt**TAAAGT**agaaaa**A**TG
cj0461c	-	426422	426422	0	no	yes	taaaatataaattat**gcTATAGT**ttcgctt**A**TG
cj0595c	-	553151	553151	0	no	yes	ggtttaaagctaattaac**TAAAAT**taaaaa**A**TG
cj0641	+	602326	602326	0	no	yes	ttgctttgctaagattt**gaTATAAT**ttttt**A**TG
cj0667	+	622768	622768	0	no	yes	aaattgattttttttt**gcTAAAAT**tgctttt**A**TG
cj0846	+	793305	793305	0	no	yes	tatttaattttacttg**gtTATAAT**actttt**A**TG
cj1031	+	963479	963479	0	no	yes	aatttatataaagtatat**TAAAAT**atcaaa**A**TG
cj1152c	-	1085255	1085255	0	no	yes	tttttaaaaaagctatat**TATAAT**gttttt**A**TG
cj1247c	-	1176327	1176327	0	no	yes	aattaagaaaagaaat**gcTAAAAT**catctt**A**TG
cj1257c	-	1188360	1188360	0	no	yes	aaattataaaaaatatca**TACAAT**aaatcc**A**TG
cj1349c	-	1282299	1282299	0	no	yes	aattcgccaaaatattt**TATAAT**ttctttt**A**TG
cj1565c	-	1496400	1496400	0	no	yes	atgttataagagtttt**gcTATAAT**tatttt**A**TG
cj1620c	-	1548761	1548760	1	no	yes	aattttaaaattttttt**gtTATACT**ctcatt**T**ATG
cj1711c	-	1624461	1624459	2	no	yes	taattaagaattttta**gaTAAAAT**tatttc**T**tATG
cj0430	+	391708	391711	3	no	yes	atattttaagtttaattttg**TAAAAT**tttc**A**ttATG
cj1449c	-	1388187	1388184	3	no	yes	tttttaaaaatattttag**TATAAT**ttttac**A**atATG
cj1458c	-	1396196	1396193	3	no	yes	aatttaaaaggaaattttta**TAAAAT**cacgc**A**ttATG
cj0792	+	742472	742477	5	no	yes	tccttataaaaaattt**gcTAAAAT**tatacta**T**aaaaATG
cj0748	+	703019	703027	8	no	yes	ataatcagtaattttttta**TAGAAT**acctat**G**cggaataATG
cj1441c	-	1380906	1380897	9	maybe	no	ttttgaggattttaatta**TAAAAT**aatgaa**AGG**taaaatATG
cj0318	+	288447	288457	10	yes	no	aaaaatatgtaaaattc**gcTAAACT**aaggg**A**t**GAGA**attaATG
cj0945c	-	886130	886119	11	yes	no	cttttagaaaaaata**gtTATAAT**taaagcc**AAGGA**aatataATG
cj1638	+	1562943	1562955	12	yes	no	attatagccaaatctttg**TATAAT**tttaac**AAGG**tttgtaaaATG
cj0829c	-	776786	776773	13	yes	no	ttttttaatcatttaa**gaTAAAAT**accaaa**A**ca**AAGG**ttaacaATG
cj1350	+	1282348	1282361	13	yes	no	ttttggcgaattttgt**ggTAGAAT**tacatt**A**aaa**AGGA**aatttGTG
cj1618c	-	1546343	1546330	13	yes	no	aaatttcataaattttt**ggTAAAAT**gtttac**T**ct**AGGA**aatataATG

a) Genes are sorted on length of the 5' UTR.

b) Capital letters indicate relevant motifs: promoters (bold, red typeface for the σ^{70} promoters with TAnaaT or gnTAnaaT motifs), TSS (green, underlined and bold typeface), putative ribosome binding site (blue, bold typeface) and translational startcodon.
