# Supplementary material for: Parallel evolution of genome structure and transcriptional landscape in the Epsilonproteobacteria
Source: BMC Genomics. 2013 Sep 12;14:616. doi: 10.1186/1471-2164-14-616 (PMC3847290; doi:10.1186/1471-2164-14-616)
Supplement: Additional file 14: Table S7 — Epsilonproteobacterial and other genome sequences used in this study. [file 1471-2164-14-616-S14.pdf]

Table S7. Epsilon-proteobacterial and other genome sequences used in this study

Species	strain / isolate	Proteobacteria	Accession number
Arcobacter butzleri	RM4018	Epsilon	NC_009850.1
Arcobacter nitrofigilis	DSM 7299	Epsilon	NC_014166.1
Arcobacter sp.	L	Epsilon	AP012048.1
Caminibacter mediatlanticus	TB-2	Epsilon	NZ_ABCJ00000000
Campylobacter coli	RM2228	Epsilon	NZ_AAFL00000000
Campylobacter concisus	13826	Epsilon	NC_009802.1
Campylobacter curvus	52592	Epsilon	NC_009715.1
Campylobacter fetus subsp *fetus*	82-40	Epsilon	NC_008599.1
Campylobacter gracilis	RM3268	Epsilon	NZ_ACYG00000000
Campylobacter hominis	ATCC BAA-381	Epsilon	NC_009714.1
Campylobacter jejuni	NCTC 11168	Epsilon	NC_002163.1
Campylobacter jejuni	RM1221	Epsilon	NC_003912.7
Campylobacter jejuni	81-176	Epsilon	NC_008787.1
Campylobacter jejuni subsp *doylei*	26997	Epsilon	NC_009707.1
Campylobacter lari	RM2100	Epsilon	NC_012039.1
Campylobacter rectus	RM3267	Epsilon	NZ_ACFU00000000
Campylobacter showae	RM3277	Epsilon	NZ_ACVQ00000000
Campylobacter upsaliensis	RM3195	Epsilon	NZ_AAFJ00000000
Escherichia coli K-12	substrain MG1655	**Gamma**	NC_000913
Helicobacter acinonychis	Sheeba	Epsilon	NC_008229.1
Helicobacter bilis	ATCC 43879	Epsilon	NZ_ACDN00000000
Helicobacter bizzozeroni	CIII	Epsilon	NC_015674.1
Helicobacter canadensis	MIT 98-5491	Epsilon	NZ_CM000776.1
Helicobacter cetorum	MIT99-5656	Epsilon	CP003481.1
Helicobacter cinaedi	PAGU611	Epsilon	AP012344
Helicobacter felis	ATCC 49179	Epsilon	NC_014810.1
Helicobacter hepaticus	ATCC 51449	Epsilon	NC_004917.1
Helicobacter mustelae	12198	Epsilon	NC_013949.1
Helicobacter pullorum	MIT 98-5489	Epsilon	NZ_ABQU00000000
Helicobacter pylori	J99	Epsilon	NC_000921.1
Helicobacter pylori	26695	Epsilon	NC_000915.1
Helicobacter pylori	HPAG1	Epsilon	NC_008086.1
Helicobacter suis	HS1	Epsilon	NZ_ADGY00000000
Helicobacter winghamensis	ATCC BAA-430	Epsilon	NZ_ACDO00000000
Nautilia profundicola	Am-H	Epsilon	NC_012115.1
Nitratifractor salsuginis	DSM 16511	Epsilon	NC_014935.1
Nitratiruptor sp.	SB155-2	Epsilon	NC_009662.1
Sulfuricurvum kujiense	DSM 16994	Epsilon	NC_014754.1
Sulfurimonas autotrophica	DSM 16294	Epsilon	NC_014506.1
Sulfurimonas denitrificans	DSM 1251	Epsilon	NC_007575.1
Sulfurimonas gotlandica	GD1	Epsilon	NZ_AFRZ00000000
Sulfurospirillum barnesii	SES3	Epsilon	CP003333.1
Sulfurospirillum deleyianum	DSM 6946	Epsilon	NC_013512.1
Sulfurospirillum sp.	Am-N	Epsilon	PRJNA46505
Sulfurovum sp.	NBC37-1	Epsilon	NC_009663.1
Sulfurovum sp.	AR	Epsilon	NZ_AJLE00000000
Thiomicrospira crunogena	XCL-2	**Gamma**	NC_007520.2
Wolinella succinogenes	DSM 1740	Epsilon	NC_005090.1
