# Supplementary material for: Parallel evolution of genome structure and transcriptional landscape in the Epsilonproteobacteria
Source: BMC Genomics. 2013 Sep 12;14:616. doi: 10.1186/1471-2164-14-616 (PMC3847290; doi:10.1186/1471-2164-14-616)
Supplement: Additional file 15: Figure S8 — Lack of gene order-based genome synteny in the Epsilonproteobacteria. All protein-coding annotated features of 42 species of Epsilonproteobacteria (Additional file 14: Table S7) were compared by pairwise BLASTP against C. jejuni NCTC 11168 (pages 1–2) and H. pylori 26695 (pages 3–4). The highest scoring ortholog in the pairwise comparison was used if the E-score was > 1 E-06, and used in a scatter plot [77]. The Gamma-proteobacteria E. coli and Thiomicrospora crunogena are included for comparison. An overview of the total number of genes orthologous between these species is given in Additional file 16: Table S8. [file 1471-2164-14-616-S15.pdf]

Pairwise genome synteny comparison of *C. jejuni* NCTC 11168 with:

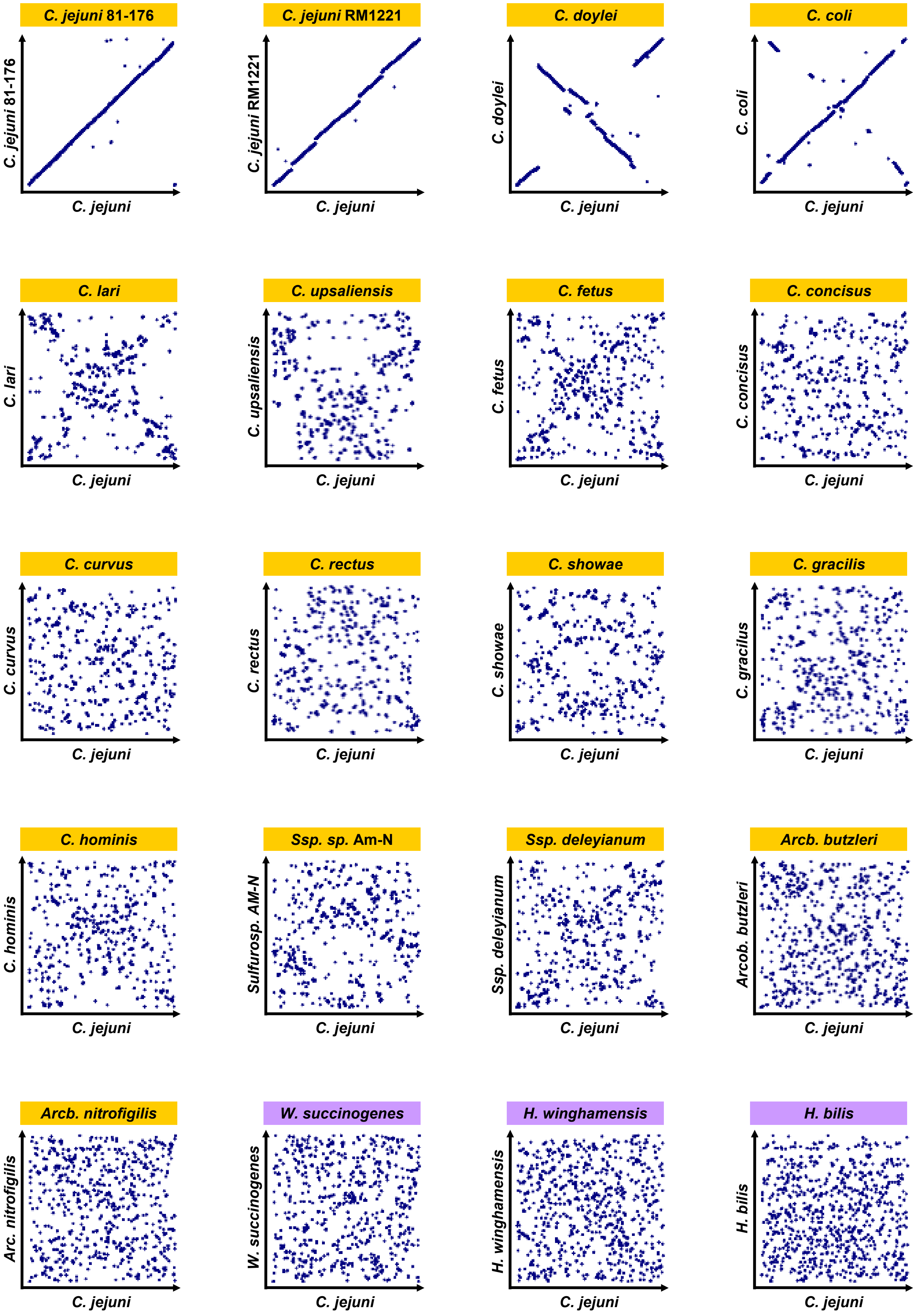

Porcelli et al, Figure S8, part 1 of 4

Pairwise genome synteny comparison of *C. jejuni* NCTC 11168 with:

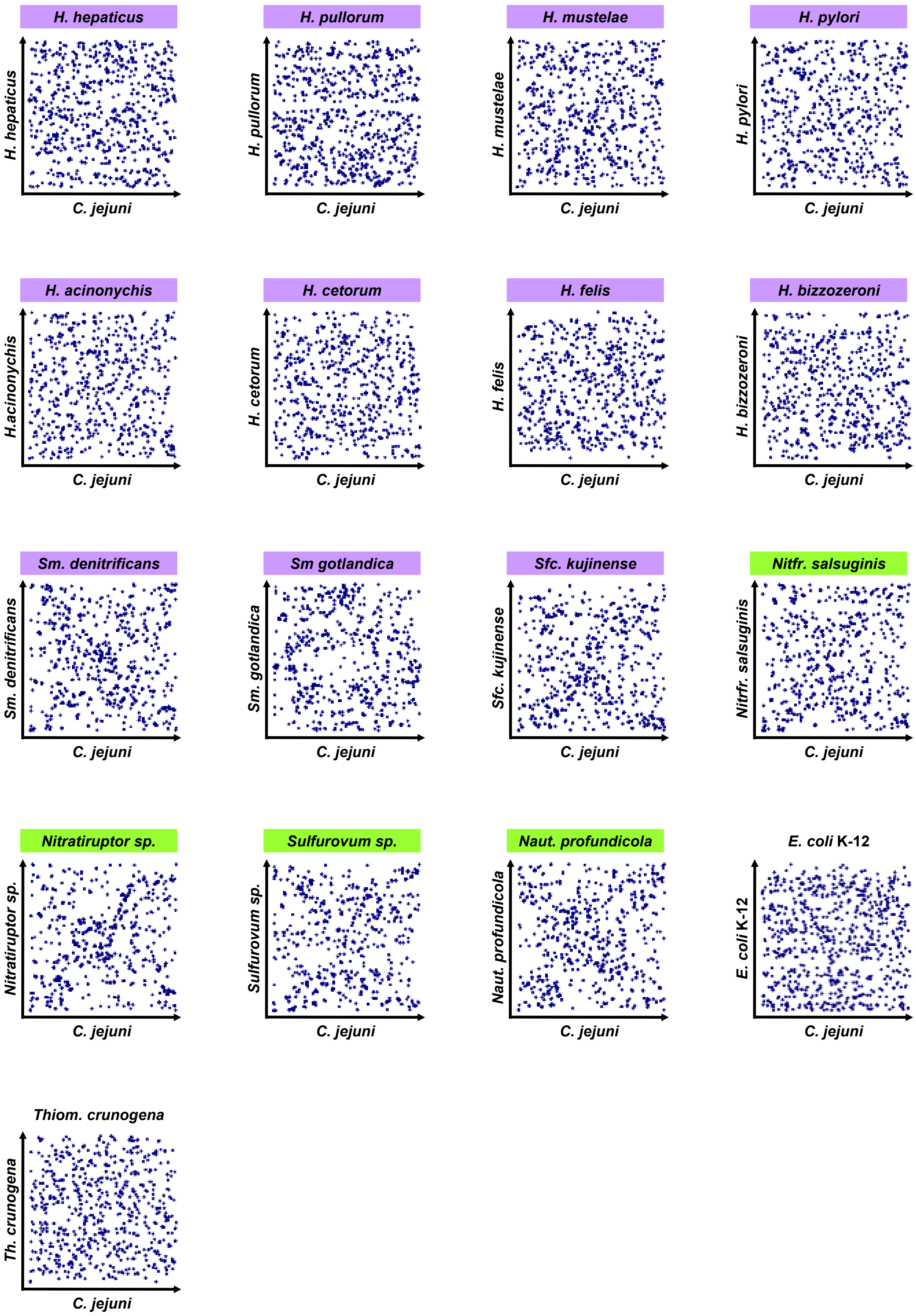

Porcelli et al, Figure S8, part 2 of 4

Pairwise genome synteny comparison of *H. pylori* 26695 with:

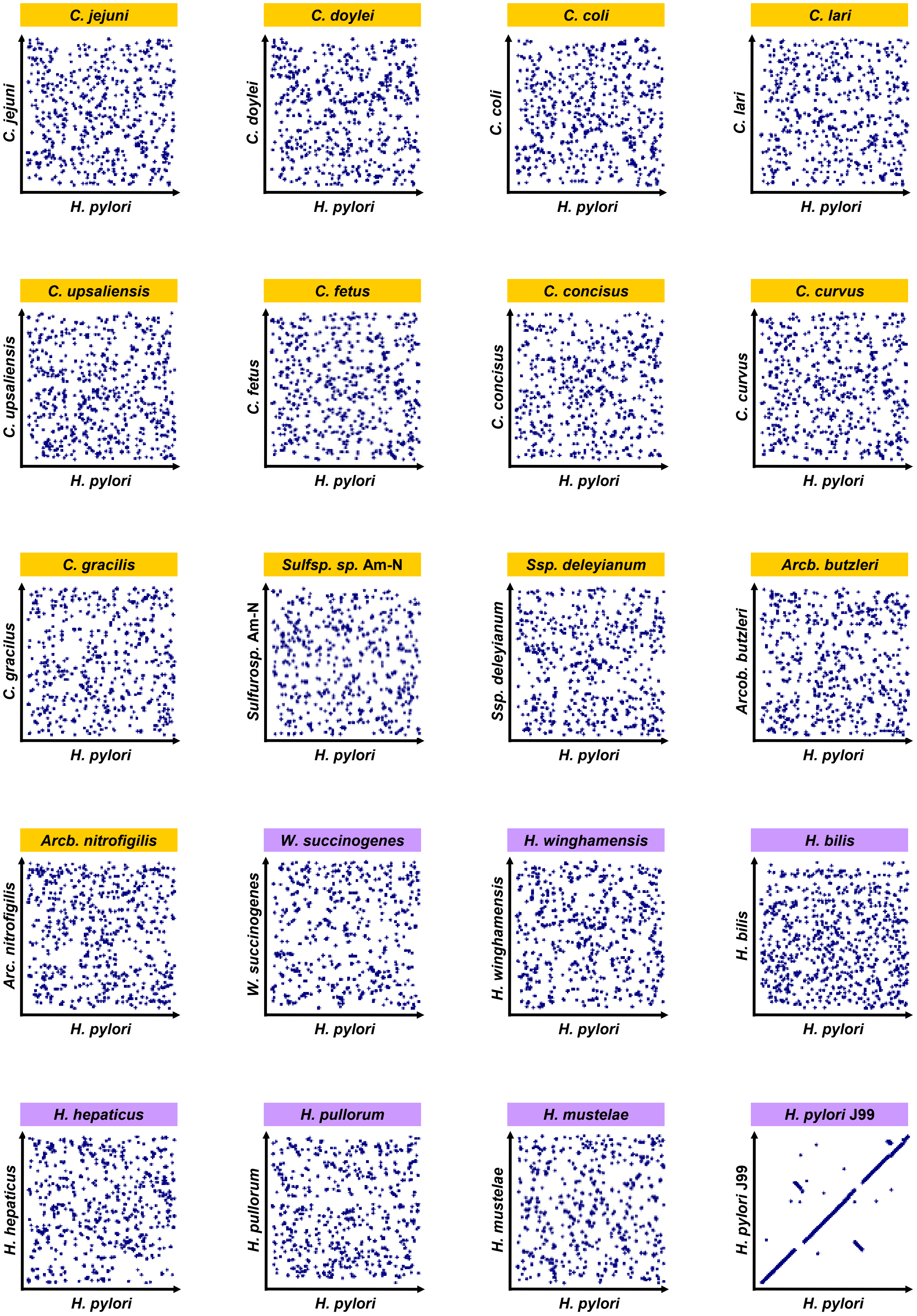

Porcelli et al, Figure S8, part 3 of 4

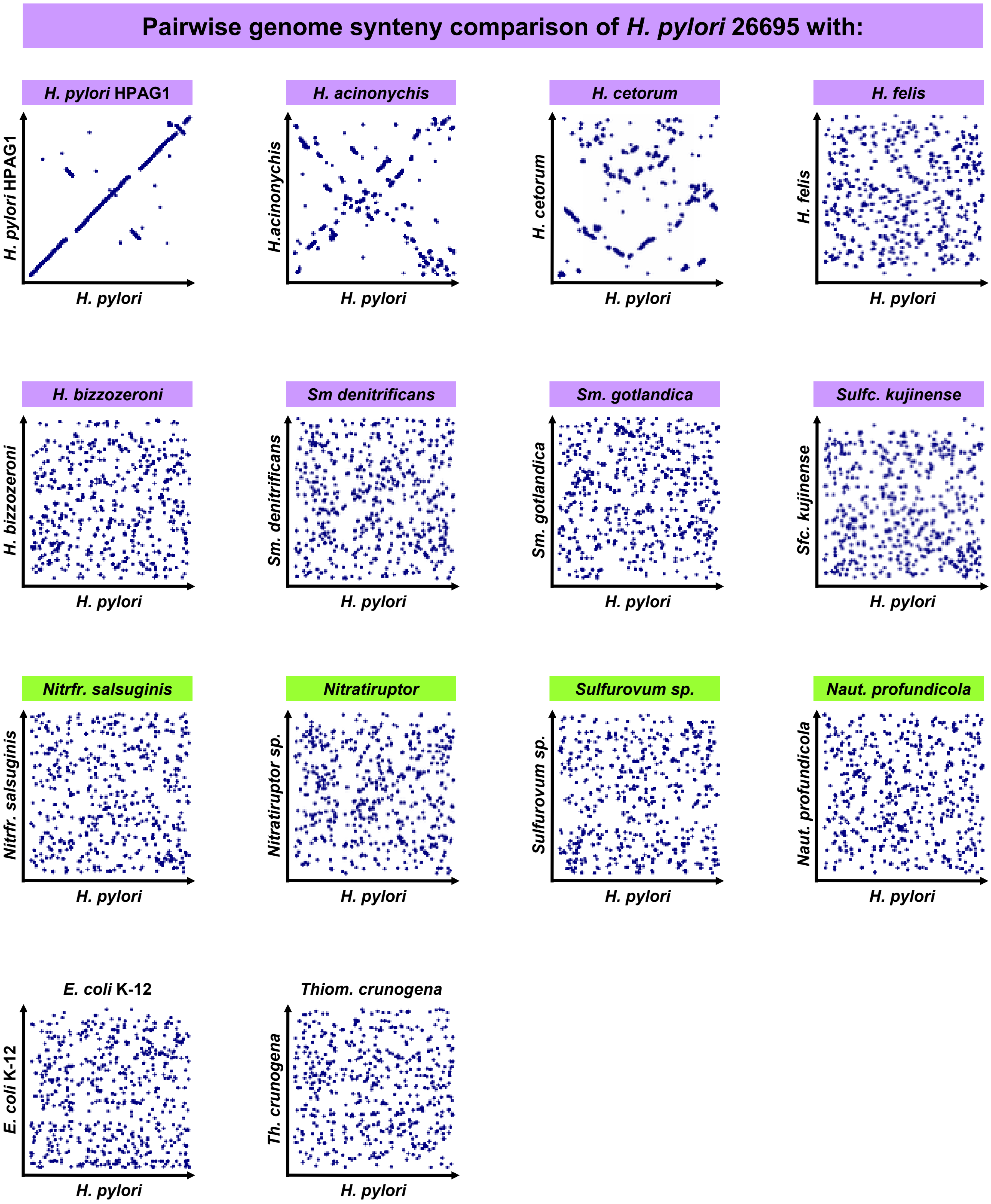

Porcelli et al, Figure S8, part 4 of 4
