# Supplementary material for: Parallel evolution of genome structure and transcriptional landscape in the Epsilonproteobacteria
Source: BMC Genomics. 2013 Sep 12;14:616. doi: 10.1186/1471-2164-14-616 (PMC3847290; doi:10.1186/1471-2164-14-616)
Supplement: Additional file 22: Table S13 — Oligonucleotides used in this study. [file 1471-2164-14-616-S22.pdf]

Table S13. Oligonucleotides used in this study

Primer name	Nucleotide sequence (5' → 3')
454-FW [a]	GCCTCCCTCGCGCCATCAGCTNNNNGACCTTGGCTGTCACTCA
454-REV-polyT	GCCTTGCCAGCCCGCTCAGACGAGACATCGCCCCGCTTTTTTTTTTTTTTTTTTTTTTTT
RNA adapter	AUAUGCGCGAAUUCCUGUAGAACGAACACUAGAAGAAA
5’ adapter primer	GCGCGAATTCCTGTAGA
NC1REV	GGCAAATTGCAATATTTTGAA
NC3REV	GTAGGCGTGTAATTTTAAGGA
NC4REV	TCATGGGCTTGATTGCAAT
NC5REV	TCAGGATGAAATTTTTAACAAGTAGG
NC8REV	GGAAATAGCCTAACCCAAACG
NC9REV	TTCCCTAAGTCAAGCCTTTCA
NC10REV	TTCTGAGCCCATTTCGATCT
6SREV	GCTGCAACTTCTCGCTTTTT
5SREV	TACTTTCCCCCTGCCAGTAA
SRPRNAREV	GCGGCACACACTAAAATCAA
RNA adaptor	AUAUGCGCGAAUUCCUGUAGAACGAACACUAGAAGAAA
RNA Adaptor-primer	GCGCGAATTCCTGTAGA
Cj0008_antisense	GAATCCATAGAAGCTAAATG
Cj0671_antisense	TCAAGCAGCGGCTATTTCAG
Cj1355c_antisense	GTGAAGGATAGTCTAGGAGA
Cj1615_antisense	GCGGAGATGATGAAGCTAGA
Cj0696_internal	ATTCATAATTTTATGCCAGA
Cj1103_internal_1	AATAATCTCCTTTATCATGA
Cj1103_internal_2	ATATCATTCTGAACAACAGA

a) The NNNN in the primer was used for barcoding libraries. The CJE+ library used barcode GTAT, the CJE- library used GCTC.
